# Inordinate Spinescence: Taxonomic Revision and Microtomography of the *Pheidole cervicornis* Species Group (Hymenoptera, Formicidae)

**DOI:** 10.1371/journal.pone.0156709

**Published:** 2016-07-27

**Authors:** Eli M. Sarnat, Georg Fischer, Evan P. Economo

**Affiliations:** 1 Okinawa Institute of Science & Technology Graduate University, 1919–1 Tancha, Onna-son, Okinawa, 904–0495, Japan; 2 Department of Ecology and Evolutionary Biology, University of Michigan, Ann Arbor, Michigan, United States of America; University of Innsbruck, AUSTRIA

## Abstract

The ant genus *Pheidole*—for all of its hyperdiversity and global ubiquity—is remarkably conservative with regard to morphological disparity. A striking exception to this constrained morphology is the spinescent morphotype, which has evolved multiple times across distantly related lineages of Indoaustralian *Pheidole*. The *Pheidole cervicornis* group contains perhaps the most extraordinary spinescent forms of all *Pheidole*. Here we present a taxonomic revision of the *P*. *cervicornis* group, and use microtomographic scanning technology to investigate the internal anatomy of the thoracic spines. Our findings suggest the pronotal spines of *Pheidole* majors, are possibly skeletomuscular adaptations for supporting their disproportionately large heads. The ‘head support hypothesis’ is an alternative to the mechanical defense hypothesis most often used to explain spinescence in ants. The *P*. *cervicornis* group is known only from New Guinea and is represented by the following four species, including two described here as new: *P*. *barumtaun* Donisthorpe, *P*. *drogon* sp. nov., *P*. *cervicornis* Emery, and *P*. *viserion* sp. nov. The group is most readily identified by the minor worker caste, which has extremely long pronotal spines and strongly bifurcating propodeal spines. The major and minor workers of all species are illustrated with specimen photographs, with the exception of the major worker of *P*. *cervicornis*, which is not known.

## Introduction

The global rise of the hyperdiverse ant genus *Pheidole* is an evolutionary epic with many subplots [1, 2]. One of the most intriguing of these is the evolution of highly adorned “spinescent” *Pheidole*. This extreme morphotype is represented by a small minority of spiny outliers within a genus not otherwise known for morphological disparity [3, 4]. Although the genus originated in the Americas, the massive radiation of New World *Pheidole* do not include any extant spinescent species. Rather, spinescent *Pheidole* are enigmatically confined to the Old World Indo-Australian tropics. The only exceptions to this rule are the fossil taxa *P*. *tethepa* Wilson and *P*. *primigenia* Baroni Urbani described from Dominican amber [5, 6]. Once classified under the single subgenus *Pheidolacanthinus*, recent phylogenetic studies revealed that spinescent *Pheidole* evolved independently in four independent Indoaustralian lineages [1, 7]. The evolution of spinescent *Pheidole* and associated ecological changes has been studied in the context of evolutionary biogeography and the taxon cycle hypothesis [7–9]. The ecological significance of spinescence, however, remains unexplained.

Here we present our research on the most spinescent of all 1000+ *Pheidole* known to science. We used 3D X-ray microtomography to illustrate these extreme phenotypes, and to investigate the functional morphology of spinescence. Micro-ct is an emerging tool for taxonomic (e.g. [10, 11]) and functional anatomy studies (e.g. [12]). We used microtomography to test the hypothesis that pronotal spines in *Pheidole* majors are a skeletomuscular adaptation for supporting disproportionately large heads. While the oversized heads and massive mandibles of *Pheidole* majors can serve as effective colony defense, they are used by many species as biological millstones for processing seeds too hard for minor workers to crush [2, 13–17]. A comparative study of thorax architecture among ants recently revealed that, in contrast to queens, workers possess enlarged neck muscles in the first thoracic (T1) segment [18]. These expanded thoracic muscles are the modification that allows worker ants to lift and carry objects many times heavier than themselves. Presumably, the T1 muscles also allow *Pheidole* majors to support their massive head capsules and dense mandibles. If the thoracic muscles extend into the pronotal spine cavity, the extra capacity and transverse angle could increase head support. Alternatively, if the pronotal spines are devoid of muscle fibers, their function is unrelated to head support.

The subject of this study, the *Pheidole cervicornis* group (Formicidae: Myrmicinae), consists of four remarkably spinescent species (two of which are described here for the first time) that are endemic to mid-and high-elevation forests of New Guinea. The group was erected by Emery [19] to house the single species *P*. *cervicornis* Emery. Wilson [20] and Sarnat [21] both proposed that the *cervicornis* group was closely related to the spinescent *Electropheidole* (= *Pheidole roosevelti* group) of Fiji. However, subsequent phylogenetic analyses demonstrated that these two groups are only distantly related to each other, and that spinescence has evolved independently in each species clade [1, 7]. The *cervicornis* group is putatively monophyletic [1] and sister to a much larger radiation of less spinescent species in the *P*. *bifurca* group. These two sister groups are descended from a major clade of *Pheidole* that have radiated throughout New Guinea and Australia.

The following study is a small contribution towards understanding the evolutionary landscape that precipitated a handful of distantly related *Pheidole* lineages to converge towards a shared phenotype of extreme spinescence. Unlike the celebrated *Anolis* lizards of the Caribbean [22] and the Cichlid fishes of African lakes [23]—or even the spiders of Hawaii [24]—a substantial amount of work remains to be done on the basic taxonomy, morphology and ecology of Indoaustralian *Pheidole* before sufficient data is available for testing theories of convergent evolution, adaptive radiation, and morphological innovation. In the future, we hope to extend the revisionary and anatomical work presented here for the *P*. *cervicornis* group to the other major radiations of spinescent *Pheidole*.

## Methods

### X-ray microtomography

X-ray microtomography scans were taken with a Zeiss Xradia 510 Versa scanner and the included XMController software. XMReconstructer software was used post-imaging for 3D reconstruction and output files were saved in dicom format. Scanning preparation of dry mounted ant specimens on paper points only involved gluing the point including the ant to a short piece (~4cm) of MicroLumen high performance medical tubing. Parameters for all scans were chosen according to scanning directions in the Xradia 510 Versa manual. Images were taken with a 4x objective, binning of two by two pixels. Other scan parameters typically varied from 30 to 65 keV, 2 to 5.5 W. Exposure times were between 1 and 15 seconds (Table 1). Full 360 degree rotations were done with 1601 projections. The resulting scans have a resolution of typically 1013x992x993 (HxWxD) pixels and voxel sizes range from 0.00286 to 0.00549 mm.

**Table 1:** Data summary for all reported micro-CT scanned specimens.

Species	Specimen code	Subcaste	File type	Voltage (keV)	MicroA	Power (W)	Exposure (s)	Source distance (mm)	Detector distance (mm)	Voxel size/slice thickness
***P*. *barumtaun***	CASENT0709598	major	dicom	65	84	6	3	65	15	5.49E-03
***P*. *barumtaun***	CASENT0741213	minor	dicom	40	75	3	1	14.01	10.01	3.89E-03
***P*. *cervicornis***	CASENT0282330	minor	dicom	30	66	2	10	27.89	37.93	2.86E-03
***P*. *drogon***	CASENT0716380	major	dicom	40	75	3	1	13.01	12.01	3.50E-03
***P*. *drogon***	CASENT0753009	minor	dicom	40	75	3	3	30	11.01	4.93E-03
***P*. *viserion***	CASENT0219462	major	dicom	30	66	2	15	57.49	16.53	5.23E-03
***P*. *viserion***	CASENT0282331	minor	dicom	30	66	2	5	26.9	14.97	4.33E-03

Rotation videos were created using Amira software from *Volren* data visualizations by creating a *Camera Path* object with seven key frames (rotating around the three standard views: profile, full-face, and dorsal view), selecting constant rotation speed, and saving the movie files in mpeg format with 480p resolution, 24 frames per second frame rate and selecting AntiAliasing2 in the *movie maker* function. The number of mesh triangles was reduced with a three-step approach for creating the 3D PDFs. First, the freely available software Meshlab (where the scan data were imported as STL files) was used to select and manually remove the paper points bearing the specimens. The scans were cleaned from small artifacts and other isolated fragments (*Filters > Remove isolated piece (wrt diameter)* (diameter size = 1%). Second, the specimens’ interior structure was removed by selecting the functions: (1) *Filters > Color Creation and Processing > Ambient Occlusion Per Vertex*, (2) *Filters > Selection > Select Faces By Vertex Quality* (min = 0, max = 0.001), (3) *Remove Selected Faces*. This sequence isolates the visible vertexes representing the specimens’ exterior. Third, the amount of vertexes was reduced to slightly less than 1 million mesh triangles (*Filters > Quadratic Edge Collapse Decimation*). The resulting STL files, each between 200–300 megabytes, were opened in Adobe Acrobat Pro, using the Tetra4D converter app, where they were annotated and saved as 3D PDFs.

The full volumetric datasets have been archived at the Dryad Data Repository (http://datadryad.org, doi: 10.5061/dryad.1j41m).

### Morphometric measurements and illustration

External morphological characters were quantified and are reported as lengths or indices. Measurements were made with a stereomicroscope at 40x magnification using a dual-axis stage micrometer wired to digital readouts. Digital color specimen photographs were taken using the Auto-Montage software package (Syncroscopy) in combination with a JVC KY-F7U digital camera mounted on a Leica MZ16 dissecting scope, and the software package Helicon Focus in combination with a Leica DFC450 digital camera mounted on a Leica M205C dissecting scope. Vector illustrations were made in Adobe Illustrator by tracing specimen photographs.

**Fig 1:**
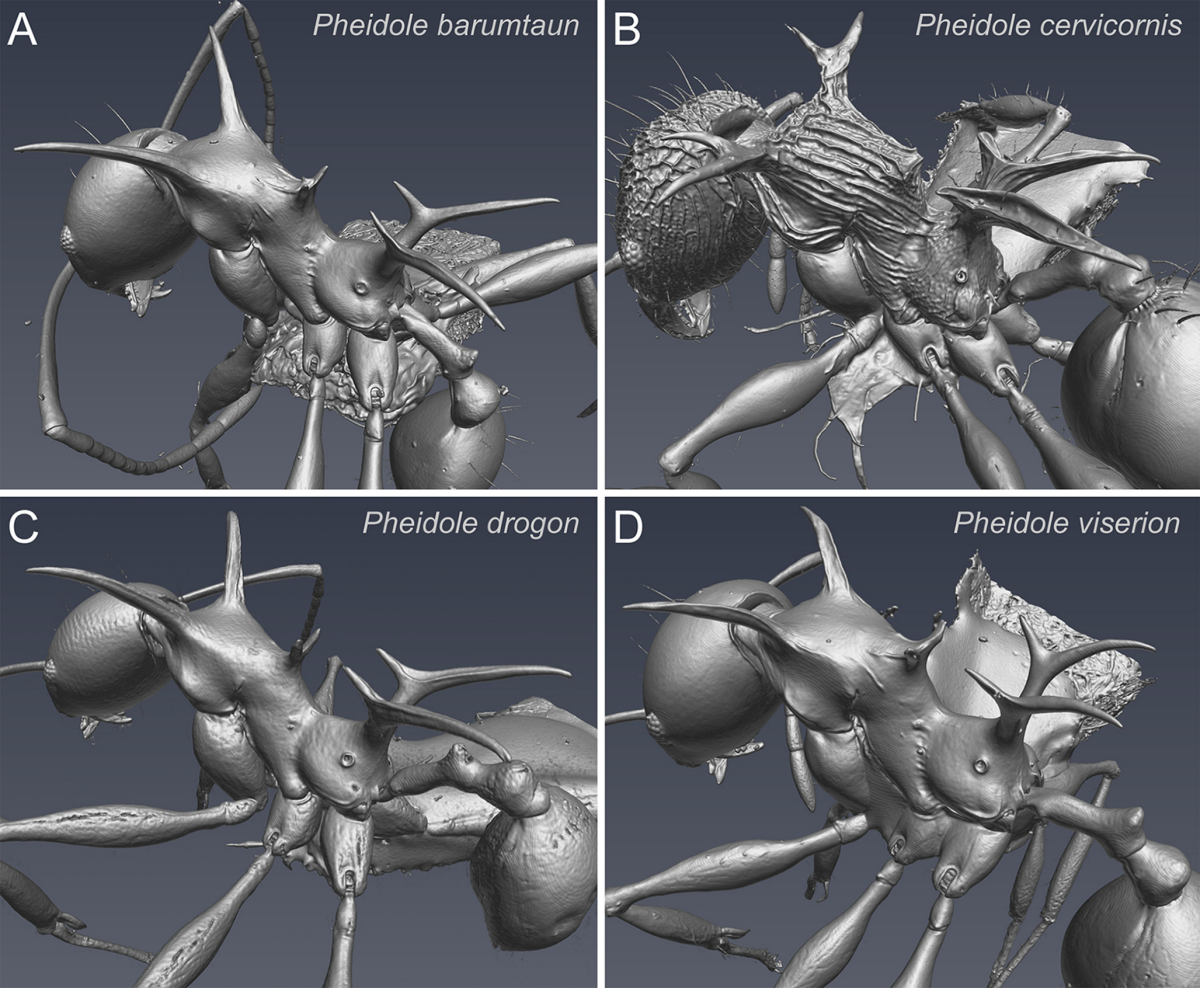
Shaded surface display volume rendering of *Pheidole cervicornis*-group minor workers. (A) *P*. *barumtaun* Donisthorpe, LACMENT316356; (B) *P*. *cervicornis* Emery, CASENT0716375; (C) *P*. *drogon* sp. nov., paratype, CASENT0753009; (D) *P*. *viserion* sp. nov., paratype, CASENT0282331.

**Fig 2:**
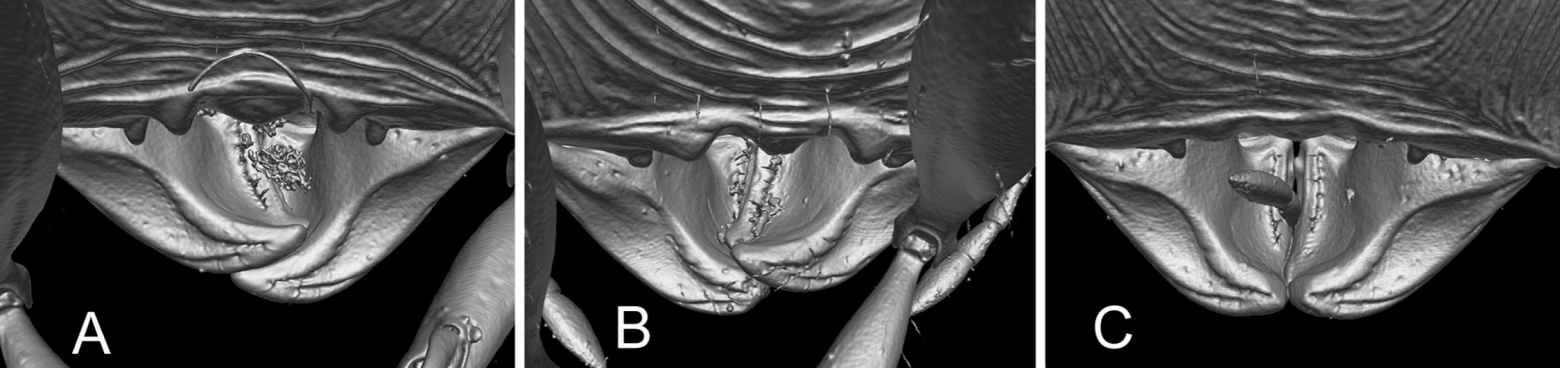
Volumetric surface model illustrating the hypostomal bridge of *Pheidole* major workers. (A) *P*. *barumtaun* Donisthorpe, CASENT0741213; (B) *P*. *drogon* sp. nov., holotype, CASENT0716380; (D) *P*. *viserion* sp. nov., holotype, CASENT0219462.

**Fig 3:**
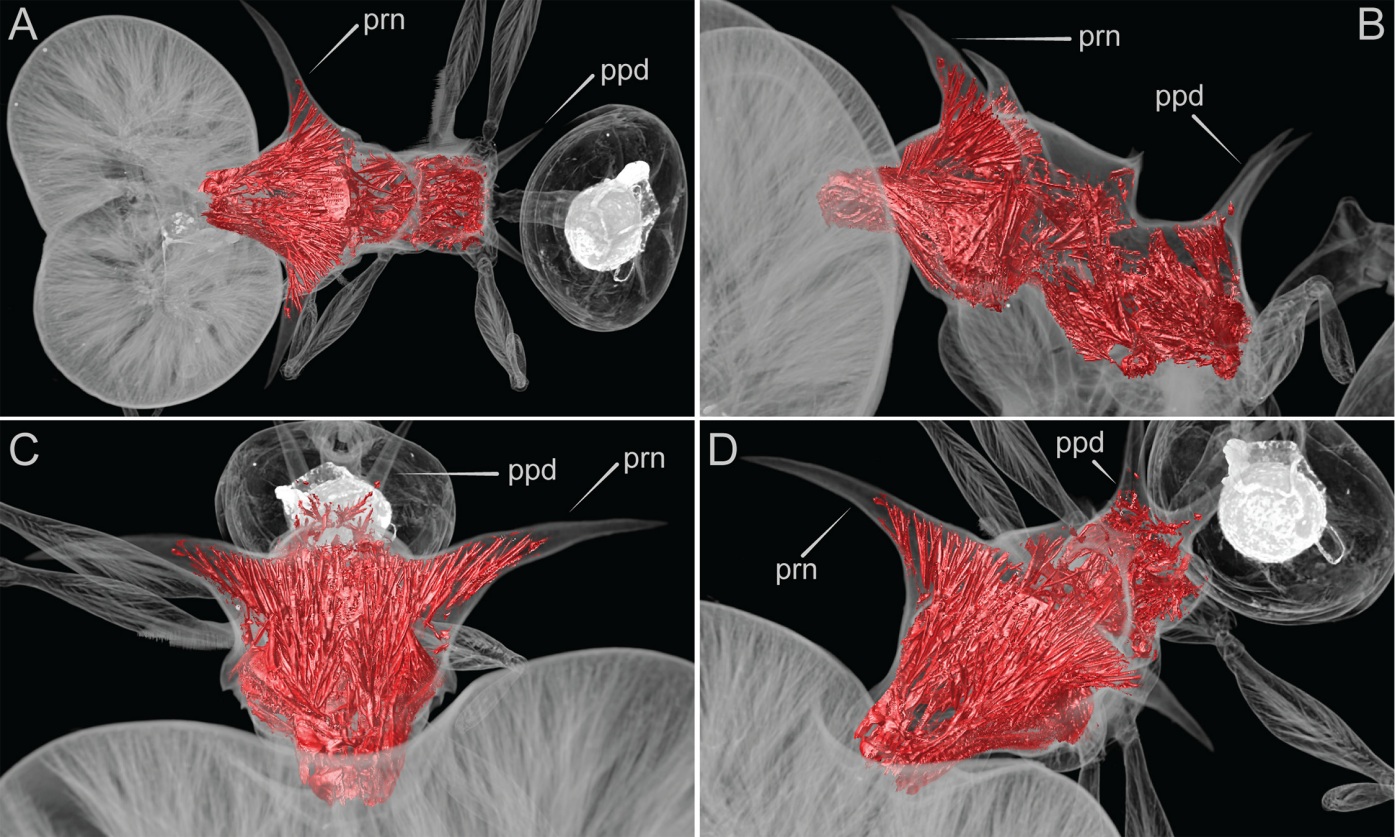
False-color volume rendering of virtually dissected mesosoma (*Pheidole drogon*, major worker, holotype, CASENT0716380). Muscle fibers are colored in red. Note the scarcity of muscle fibers in either the propodeal spines (ppd) relative to the propodeal spines (prn). (A) Dorsal view, (B) profile view, (C) anterodorsal view, (D) anterodorsal oblique view.

**Fig 4:**
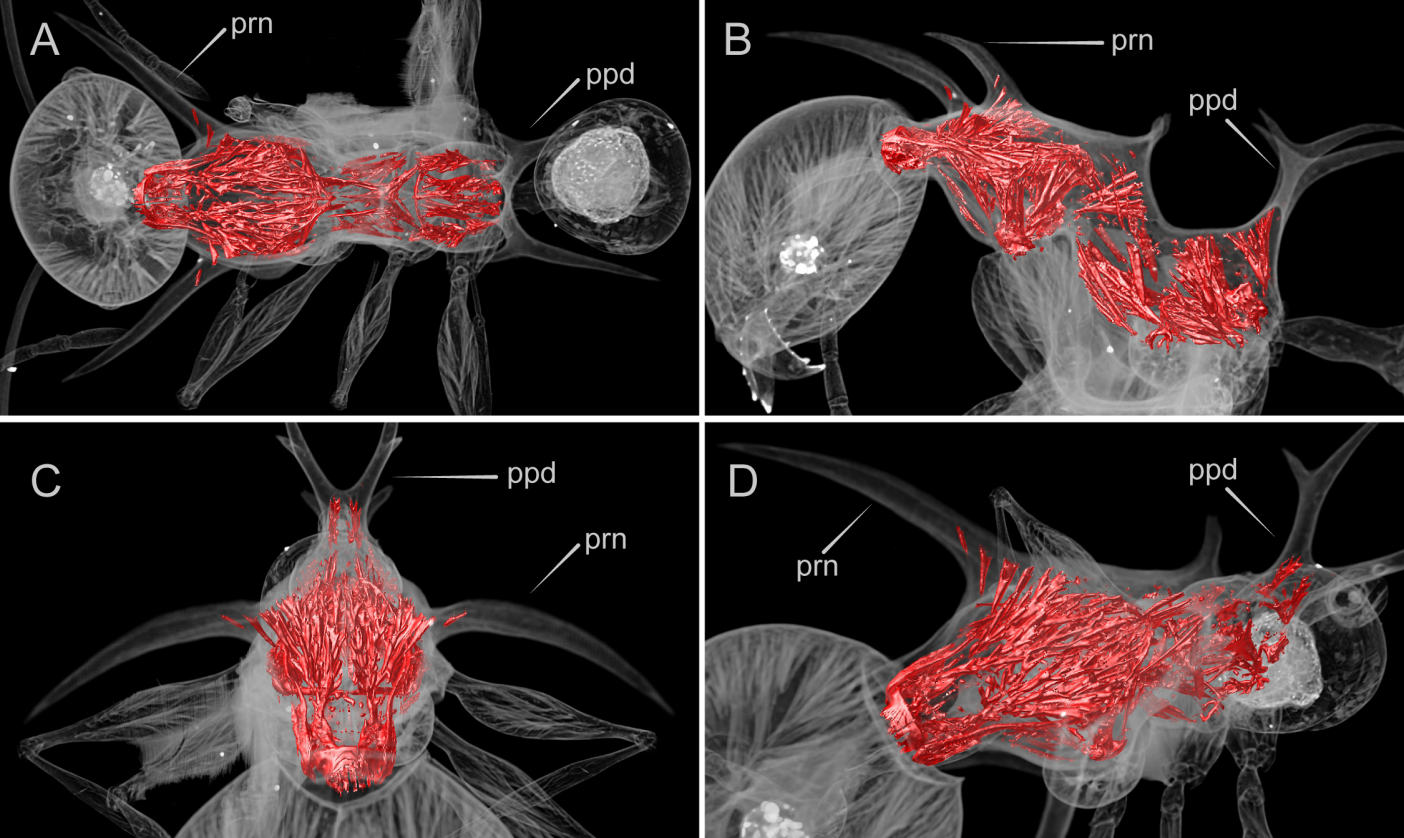
False-color volume rendering of virtually dissected mesosoma (*Pheidole drogon*, minor worker, paratype, CASENT0753009). Muscle fibers are colored in red. Note the relative scarcity of muscle fibers in either the pronotal (prn) or propodeal spines (ppd). (A) Dorsal view, (B) profile view, (C) anterodorsal view, (D) anterodorsal oblique view.

**Fig 5:**
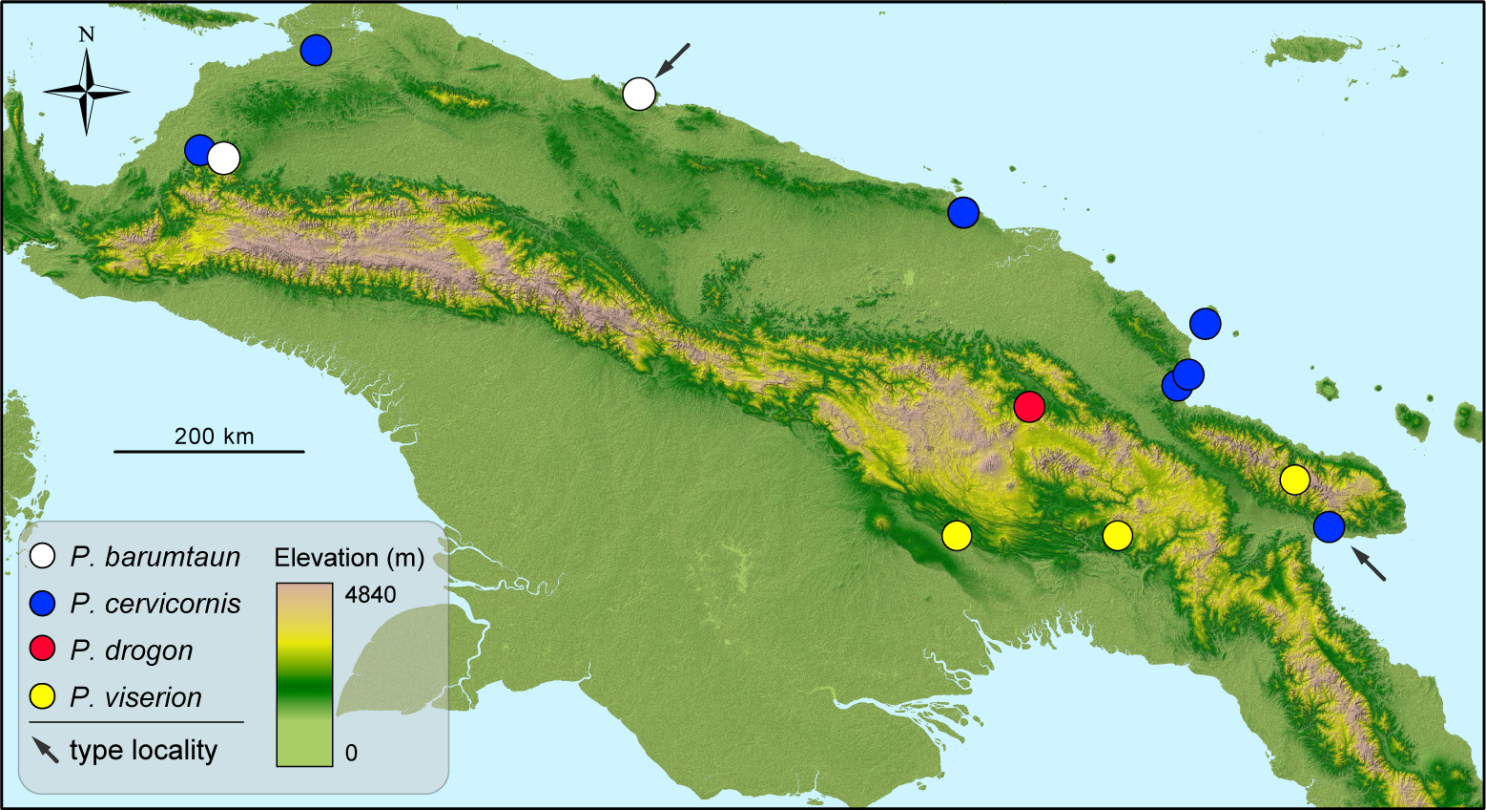
Map of New Guinea showing known occurrences of species within the *Pheidole cervicornis* group. Arrow symbols point to type localities.

**Fig 6:**
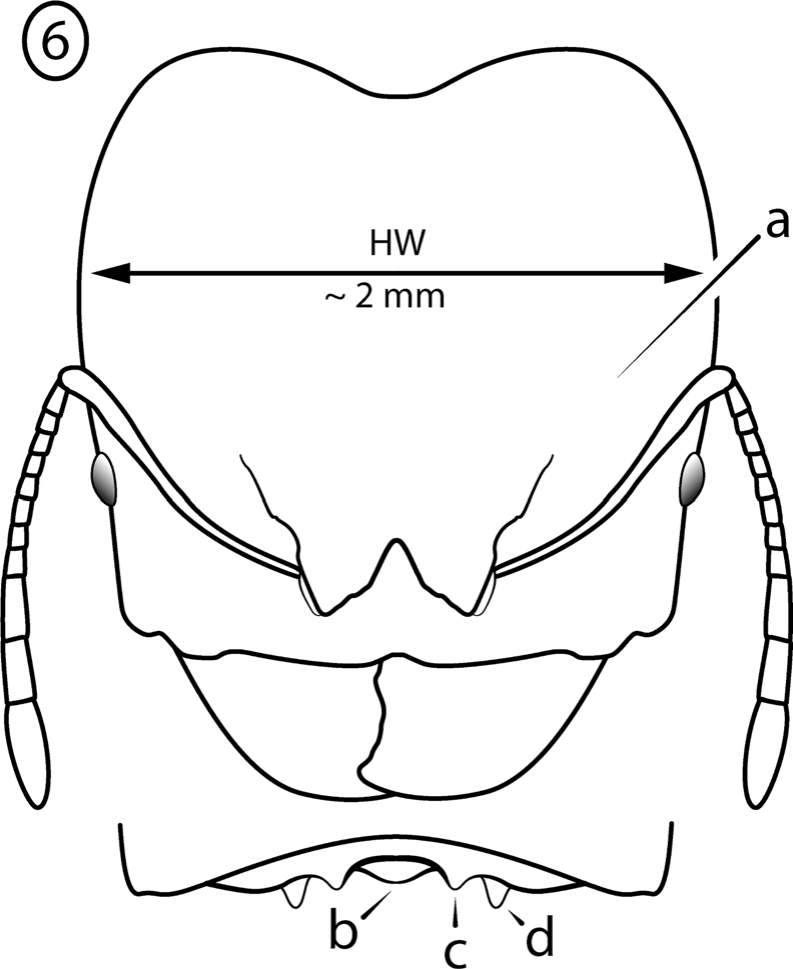
Illustration of *Pheidole cervicornis* group characteristics, head of major worker. Head Width > 1.8 mm; (a) antenna 12-segmented; (b) antennal scrobes absent. Hypostomal bridge with: (c) a single stout to moderate median tooth flanked by (d) a pair of weaker submedian teeth and (e) a pair of stout outer teeth.

**Fig 7:**
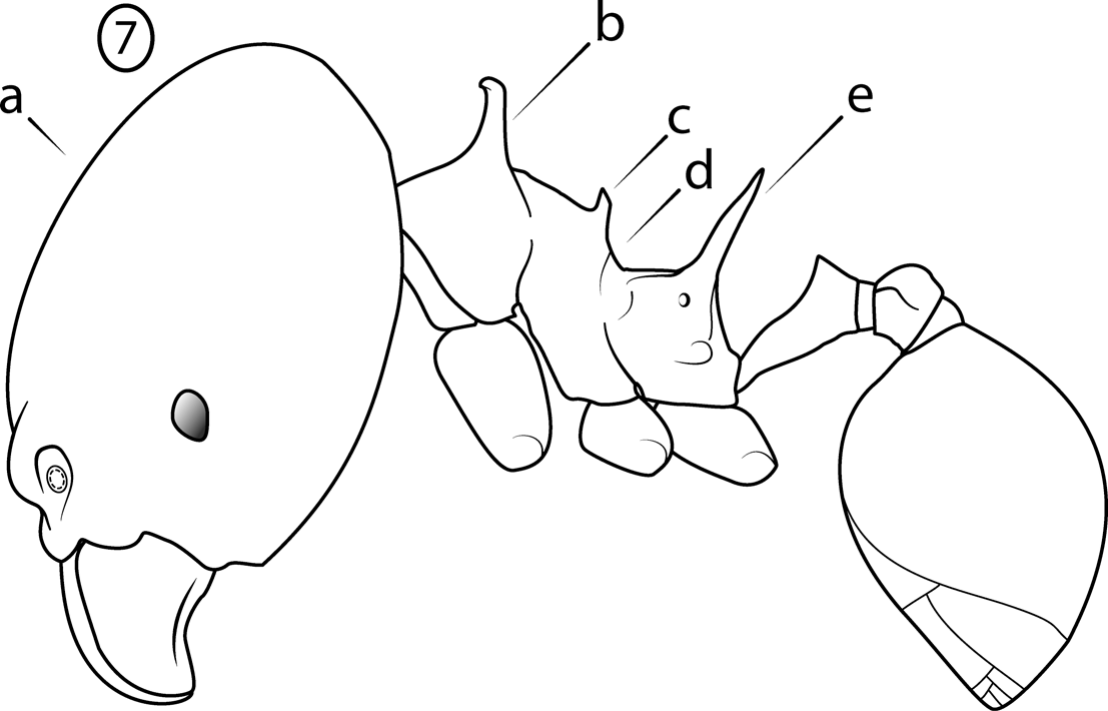
Illustration of *Pheidole cervicornis* group characteristics, profile of major worker. (a) head vertex strongly convex in profile; (b) pronotal very spines long; (c) propodeal spines very long (d) mesonotum modified with spines or lamellate lobes (e) transition from mesonotum to propodeum abrupt.

Morphometric measurements were recorded in thousandths of millimeters, but are reported here to the nearest hundredth as a range from minimum to maximum across all measured specimens. Specimens for measurements were chosen to reflect potential morphological variation across the full geographic range. The number of specimens from which measurements were taken for a given caste is referred to by *n*. Various measurements and morphological terms used here are illustrated in Sarnat (21, Figs 1–7). All specimen data and specimen images are available on the Antweb.org website, and all nomenclatural and taxonomic changes are recorded on Antcat.org website.

### Measurement and indices abbreviations

EL     *Eye length*. Maximum measureable eye length.

FL     *Metafemur length*. Length of metafemur measured along its long axis.

HL     *Head length*. Maximum distance from the midpoint of the anterior clypeal margin to the midpoint of the posterior margin of the head, measured in full-face view. In majors, measured from midpoint of tangent between anteriormost position of clypeus to midpoint of tangent between posteriormost projection of posterolateral lobes.

HW     *Head width*. Maximum width of the head in full-face view, excluding the eyes.

ML     *Mesosomal length*. Measured in lateral view as the diagonal length of the mesosoma from the point at which the pronotum meets the cervical shield to the apex of the propodeal lobe.

PeW    *Petiole width*. Maximum width of the petiole measured in dorsal view.

PeL    *Petiole length*. Maximum length of petiole measured from anteroventral junction with propodeum to posterodorsal junction with postpetiole.

PpW    *Postpetiole width*. Maximum width of the postpetiole measured in dorsal view.

PSL1   *Pronotal spine length*. Length of pronotal spine measured in dorsal-oblique view such that the base and apex of the spine are in the same focal plane. For *P*. *cervicornis* measured from middle of trunk to most apical point of posterior prong.

PSL2   *Propodeal spine length*, *vertical portion*. For major workers the length of the entire propodeal spine measured in profile view from center of propodeal spiracle to apex. For minor workers measured in profile view from the center of propodeal spiracle to the dorsal junction of the anterior and posterior projections.

PSL3   *Propodeal spine length*, *horizontal portion*. For minor workers the straight-line distance between the apices of the anterior and posterior projections of the propodeal spine, measured in profile.

SL     *Scape length*. Length of the antennal scape, including the lamella encircling the base of the scape but excluding the basal condyle.

CI     *Cephalic index*. HW/HL × 100.

SI     *Scape index*. SL/HW × 100.

### Museum abbreviations

BMNH     The Natural History Museum (London, United Kingdom)

HNHM     Hungarian Natural History Museum (Budapest, Hungary)

USNM     United States National Museum of Natural History (Washington D.C., USA)

### Nomenclatural acts

The electronic edition of this article conforms to the requirements of the amended International Code of Zoological Nomenclature, and hence the new names contained herein are available under that Code from the electronic edition of this article. This published work and the nomenclatural acts it contains have been registered in ZooBank, the online registration system for the ICZN. The ZooBank LSIDs (Life Science Identifiers) can be resolved and the associated information viewed through any standard web browser by appending the LSID to the prefix “http://zoobank.org/”. The LSID for this publication is: urn:lsid:zoobank.org:pub: 10D51A80-CD4F-4EDA-A755-FC9ADD3C81EF. The electronic edition of this work was published in a journal with an ISSN, and has been archived and is available from the following digital repositories: PubMed Central, LOCKSS [author to insert any additional repositories].

## Results and Discussion

### X-ray Microtomography

#### Taxonomic illustration, interactive 3D PDF’s and rotation videos

Volumetric surface models of the minor workers of each species (Fig 1) are presented to illustrate the extreme morphologies that have evolved within the *Pheidole cervicornis* group. Volumetric surface models of the major worker hypostomal bridge are presented to illustrate an important taxonomically and phylogenetically informative character used for diagnosing *Pheidole* clades (Fig 2). The three-dimensionality of this character is difficult to demonstrate using conventional methods, but is well-illustrated with micro-ct scans. Interactive 3D PDF’s of volumetric surface models were created for all available worker subcastes of the *Pheidole cervicornis* group species (Table 2).

**Table 2:** Output files for x-ray micro-ct scans of the *P*. *cervicornis* group specimens.

File name	Species	Subcaste	Specimen code	Type status	File type
**S1 Fig**	*P*. *barumtaun*	major	CASENT0741213	—	3d PDF
**S2 Fig**	*P*. *barumtaun*	minor	LACMENT316356	—	3d PDF
**S3 Fig**	*P*. *cervicornis*	minor	CASENT0282330	—	3d PDF
**S4 Fig**	*P*. *drogon*	major	CASENT0716380	holotype	3d PDF
**S5 Fig**	*P*. *drogon*	minor	CASENT0753009	paratype	3d PDF
**S6 Fig**	*P*. *viserion*	major	CASENT0219462	holotype	3d PDF
**S7 Fig**	*P*. *viserion*	minor	CASENT0282331	paratype	3d PDF
**S1 Vid**	*P*. *barumtaun*	major	CASENT0741213	—	3d rotation video
**S2 Vid**	*P*. *barumtaun*	minor	LACMENT316356	—	3d rotation video
**S3 Vid**	*P*. *cervicornis*	minor	CASENT0282330	—	3d rotation video
**S4 Vid**	*P*. *drogon*	major	CASENT0716380	holotype	3d rotation video
**S5 Vid**	*P*. *drogon*	minor	CASENT0753009	paratype	3d rotation video
**S6 Vid**	*P*. *viserion*	major	CASENT0219462	holotype	3d rotation video
**S7 Vid**	*P*. *viserion*	minor	CASENT0282331	paratype	3d rotation video
**S8 Vid**	*P*. *viserion*	major	CASENT0219462	holotype	virtual dissection
**S9 Vid**	*P*. *barumtaun*	major	CASENT0741213	—	orthoslice dissection

#### Internal anatomy of thoracic spines

X-ray micro-ct scans of a *Pheidole drogon* major worker specimen (Fig 3) and *P*. *viserion* (S8 Vid) reveal that the many branching muscle fibers of the first thoracic segment extend throughout substantial portions of the pronotal (anterior) spines. The pronotal spines' interior cuticle appear to serve as anchor points for muscles branches that converge anteriorly and connect to the head capsule. In contrast, the pronotal spines of the minor worker are hollow, aside from a few basal fibers (Fig 4). Moreover, the propodeal (posterior) spines of both major and minor worker of *P*. *drogon* lack substantial muscle fibers. The same musculature pattern was also observed for the other three species of the *P*. *cervicornis* group.

These findings, though preliminary, are consistent with the ‘head support hypothesis’ that pronotal spines of *Pheidole* majors are skeletomuscular adaptations for supporting their disproportionately large heads. Although a more comprehensive comparative study is necessary to rigorously test this hypothesis, these results offer the first alternative to the assumption that the spinescent architecture observed in ants functions primarily as mechanical defense

Contrary to the head support hypothesis of pronotal spines in *Pheidole* majors are the many *Pheidole* species whose majors exhibit comparably large heads but lack spinescent phenotypes. Moreover, pronotal spines are not observed in other ant lineages with specialized large-headed major workers. For example, both majors and minors of *Carebara* lack pronotal spines. Paradoxical to the head support hypothesis, pronotal spines occur in *Acanthomyrmex* minor workers but are lacking in the majors. Comparative studies, such as the one conducted by Paul and Gronenberg examining mandible muscles of ants [25], are required to further test the head support hypothesis. Future studies would benefit from the sampling of ant taxa both across spinescent and non-spinescent *Pheidole* lineages, and across other ant lineages with dimorphic worker castes.

### Distribution and diagnosis of the *Pheidole cervicornis* species group

All known occurrences of species belonging to the *Pheidole cervicornis*-group are recorded from New Guinea (Fig 5). In addition to the morphological characters common to all *Pheidole*, the following characters diagnose the worker caste of the *P*. *cervicornis* group from those of all other congeners.

Major worker: (1) Large (HW > 1.8 mm) (Fig 6); (2) Antennal scrobes absent (Fig 6A); (3) Hypostomal bridge with a single stout to moderate median tooth (Fig 6B) flanked by a pair of weaker submedian teeth (Fig 6C) and a pair of stout outer teeth (Fig 6D); (4) Head vertex strongly convex in profile (Fig 7A); (5) Pronotal spines (Fig 7B) and propodeal spines (Fig 7E) very long, subequal in length; (6) Mesonotum modified with spines or lamellate lobes (Fig 7C); (7) Transition from mesonotum to propodeum abrupt (Fig 7E).

**Fig 8:**
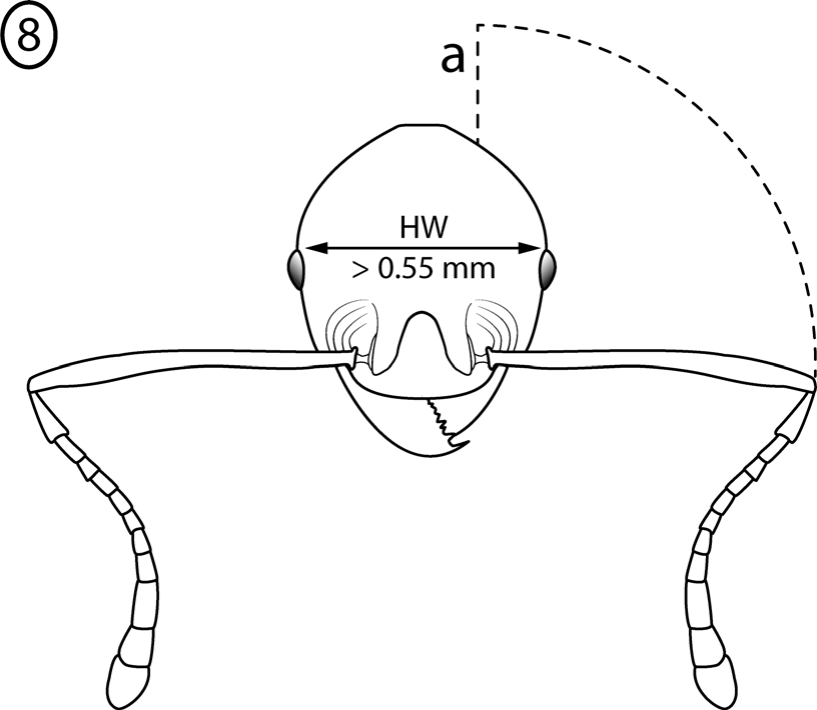
Illustration of *Pheidole cervicornis* group characteristics, head of minor worker. Head Width > 0.55 mm (a) antenna 12-segmented; (b) antennal scapes extend distinctly beyond posterior head margin.

**Fig 9:**
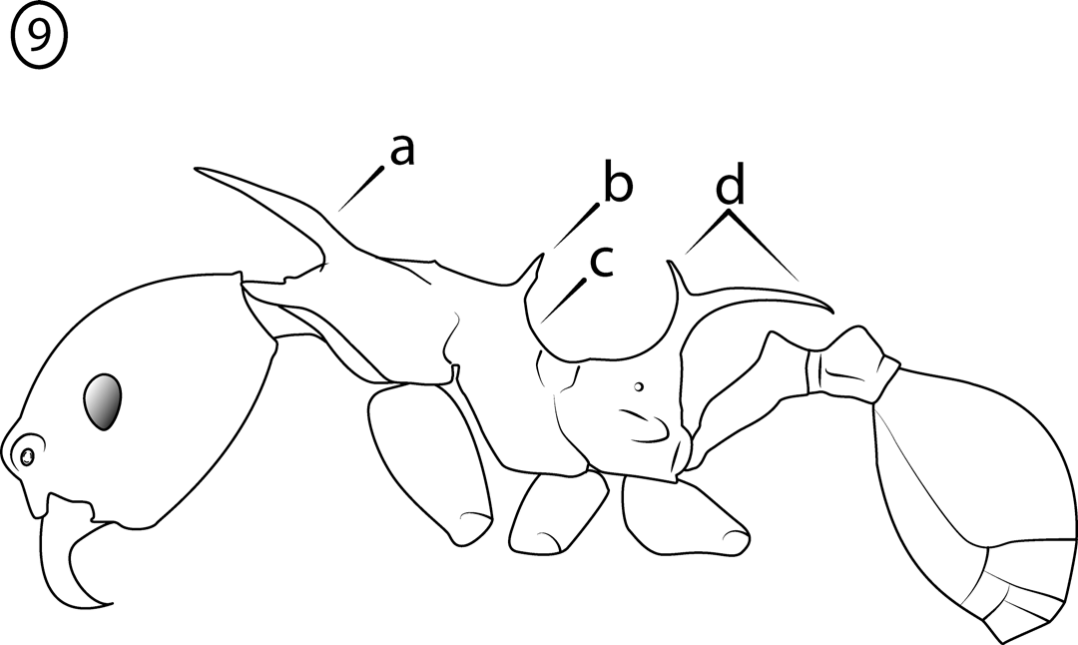
Illustration of *Pheidole cervicornis* group characteristics, profile of minor worker. (a) long pronotal spines present (simple or bifurcate); (b) mesonotum spinose (c) posterior face concave (d) propodeal spines extremely long and bifurcated.

Minor worker: (1) Large (HW > 0.55 mm) (Fig 8); (2) Antennal scape hairs suberect to erect.; (3) Antennal scapes extend distinctly beyond posterior head margin (Fig 8A); (4) Pronotal spines present, longer than eye length, subequal in length to propodeal spines, simple or bifurcate (Fig 9A); (5) Mesonotum spinose (Fig 9B), posterior face concave (Fig 9C); (6)Propodeal spines extremely long and bifurcated (Fig 9D).

All four species within the *Pheidole cervicornis* group are immediately distinguished from the vast majority of congeners by the presence of extremely long pronotal spines and propodeal spines. The only other *Pheidole* clades with pronotal spines all occur in the Old World. These include the *quadricuspis* clade, the *quadrispinosa* clade and the *bifurca* clade. The *quadricuspis* group is restricted to Southeast Asia and does not occur as far east as New Guinea. The major workers are superficially quite similar to those of the *cervicornis* group, but are distinguished by the (1) lack of a central median hypostomal tooth, (2) coarsely rugoreticulate head sculpture, and (3) shorter, thicker and more abundant pilosity. The minor workers of the *quadricuspis* group are easily distinguished by the (1) propodeal spines, which are simple and very short (subequal to eye length), and (2) lack of mesonotal spines or lobes.

The *quadrispinosa* group is sympatric with the *cervicornis* group in New Guinea. In general, *quadrispinosa* group workers are smaller than those of the *cervicornis* group, with proportionally shorter limbs. The major workers as distinguished by the (1) lack of a central median hypostomal tooth, (2) coarsely rugoreticulate head sculpture, (3) deeply excavated antennal scrobes, (4) distinctly concave head vertex, and (5) relatively shorter and more triangular pronotal spines. The minor workers are easily distinguished by their smaller size and non-bifurcating propodeal spines (although they can be strongly curved).

The *bifurca* group is reciprocally monophyletic with the *cervicornis* group, and its constituent species are unsurprisingly most difficult to distinguish from those of the *cervicornis* group. The *bifurca* group is sympatric with the *cervicornis* group (and the *quadrispinosa* group) in New Guinea. Moreover, the *bifurca* group is represented by a dizzying number of undescribed species and morphological variation. Based on our preliminary survey of the *bifurca* group, the major workers are best distinguished from those of the *cervicornis* group by the (1) shorter pronotal spines, (2) less spinose or lobate mesonotal processes, and (3) smaller size. Additionally, the hypostomal bridge of most (but not all) *bifurca* group majors have a stout median tooth and either a pair of very weak or absent submedian teeth. However, there is at least one species in which the hypostomal arrangement is similar to that of the *cervicornis* group species (stout median tooth flanked by a pair of stout submedian teeth). The minor workers of the *bifurca* group are extremely variable with respect to length and shape of their mesosomal armaments. Some have bifurcating propodeal spines, some have very long pronotal spines, and some have mesonotal processes. None that we have examined, however, possess the combination of all three. *Pheidole purpurascens* Emery perhaps comes closest to the *cervicornis* group morphotype, but this species only has angled (versus bifurcate) propodeal spines.

The only *Pheidole* species outside of the aforementioned groups that has pronotal spines are *P*. *aristotelis* Forel and *P*. *hainanensis* Chen *et al* [26, 27]. However, the pronotal and propodeal spines of both species are only subequal in length to the eye, and the latter are not bifurcated. Moreover, the major worker lacks distinct pronotal spines.

The *Pheidole roosevelti* group [21] also deserves mention here. While its constituent members lack pronotal spines, many of the species have strongly bifurcating propodeal spines that strongly resemble those seen in the *cervicornis* group, in addition to mesonotal processes. These morphological similarities are entirely convergent, however, as the *roosevelti* group is only distantly related to the *cervicornis* group [1, 7].

### Keys to the *Pheidole cervicornis* group worker subcastes

Key to major workers (major worker of *P*. *cervicornis* unknown)

**Fig 10:**
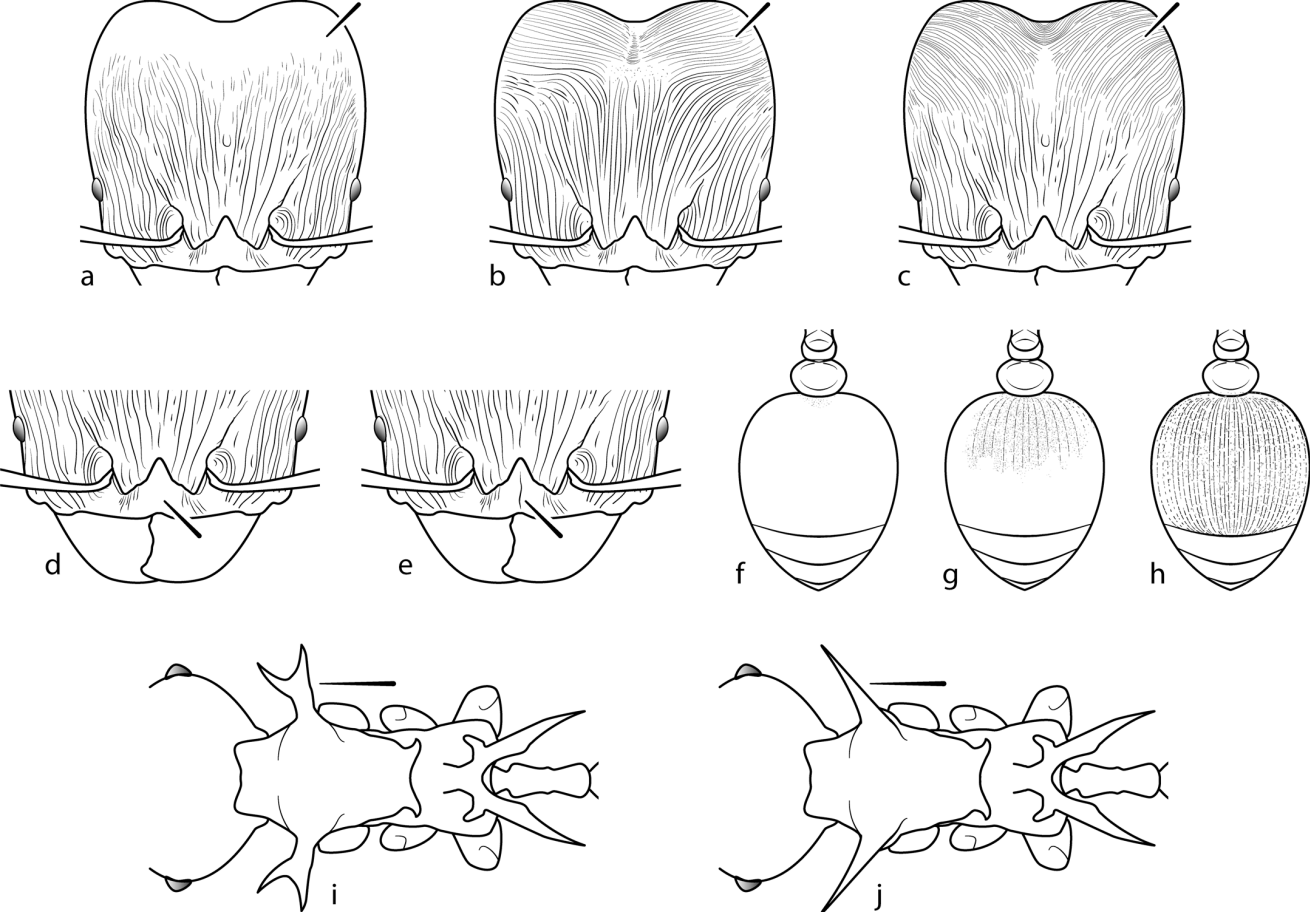
Taxonomic characters used for species diagnosis in the *Pheidole cervicornis* group.

1 Posterolateral lobes with glossy corners, free of sculpture (Fig 10A); lateral and ventral portion of lobes also glossy. Clypeus lacking strong median carina (Fig 10D). First gastral tergite entirely glossy (Fig 10F). Color uniformly dark reddish brown ***drogon***
– Corners of posterolateral lobes shining, but covered with very fine carinulae or striae (Fig 10B, Fig 10C). Clypeus with strong median carina (Fig 10E). First gastral tergite with some portion either shagreened (Fig 10G) or striate (Fig 10H), but not entirely glossy. Color variable 2
2 Head carinulae arcuate from anterior clypeal border to frons, converging towards median impression of posterolateral lobes (Fig 10B). Carinulae of posterolateral lobes distinctly transverse. Mandible with striations limited to posterolateral portion. Propodeal spines either downcurved or upturned apically. Basal third of first gastral tergite shagreened to weakly striate (Fig 10G). Color variable; some populations uniformly dark reddish brown and others with a yellowish brown mesosoma ***barumtaun***
– Head with carinulae longitudinal from anterior clypeal border to frons, then diverging laterally and becoming more fine (Fig 10C). Head carinulae of posterolateral lobes arcuate. Mandible entirely striate. Propodeal spines becoming downcurved apically. Entire first gastral tergite longitudinally striate (Fig 10H). Color uniformly yellowish brown ***viserion***

Key to minor workers

1 Pronotal armament begins as thick basal trunk that diverges into two long anterolaterally pointed prongs (Fig 10I). Head and mesosoma sculptured with thick crenulated rugulae. Head more circular (CI 94–101) ***cervicornis***
– Pronotal spines simple and non-branching (Fig 10J). All surfaces glossy, free of sculpture. Head more ovoid (CI 84–91) 2
2 Color uniformly shining yellow. Smaller (HW 0.62–0.68 mm). Antennal scapes relatively longer (SI 162–169) ***viserion***
– Color variable, but never uniformly shining yellow. Larger (HW 0.67–80 mm). Antennal scapes relatively shorter (SI 135–165) 3
3 Antennal scapes relatively longer (SI 146–165). Head narrower (CI 84–90). Legs longer (FL 1.22–1.38 mm). Color either uniformly reddish-brown, or tricolored with reddish-brown head and gaster and contrasting brownish-yellow mesosoma ***barumtaun***
– Antennal scapes relatively shorter (SI 135–141). Head more circular (CI 89–91). Legs shorter (FL 1.07–1.16 mm). Color uniformly reddish-black ***drogon***

### Species accounts

**Fig 11:**
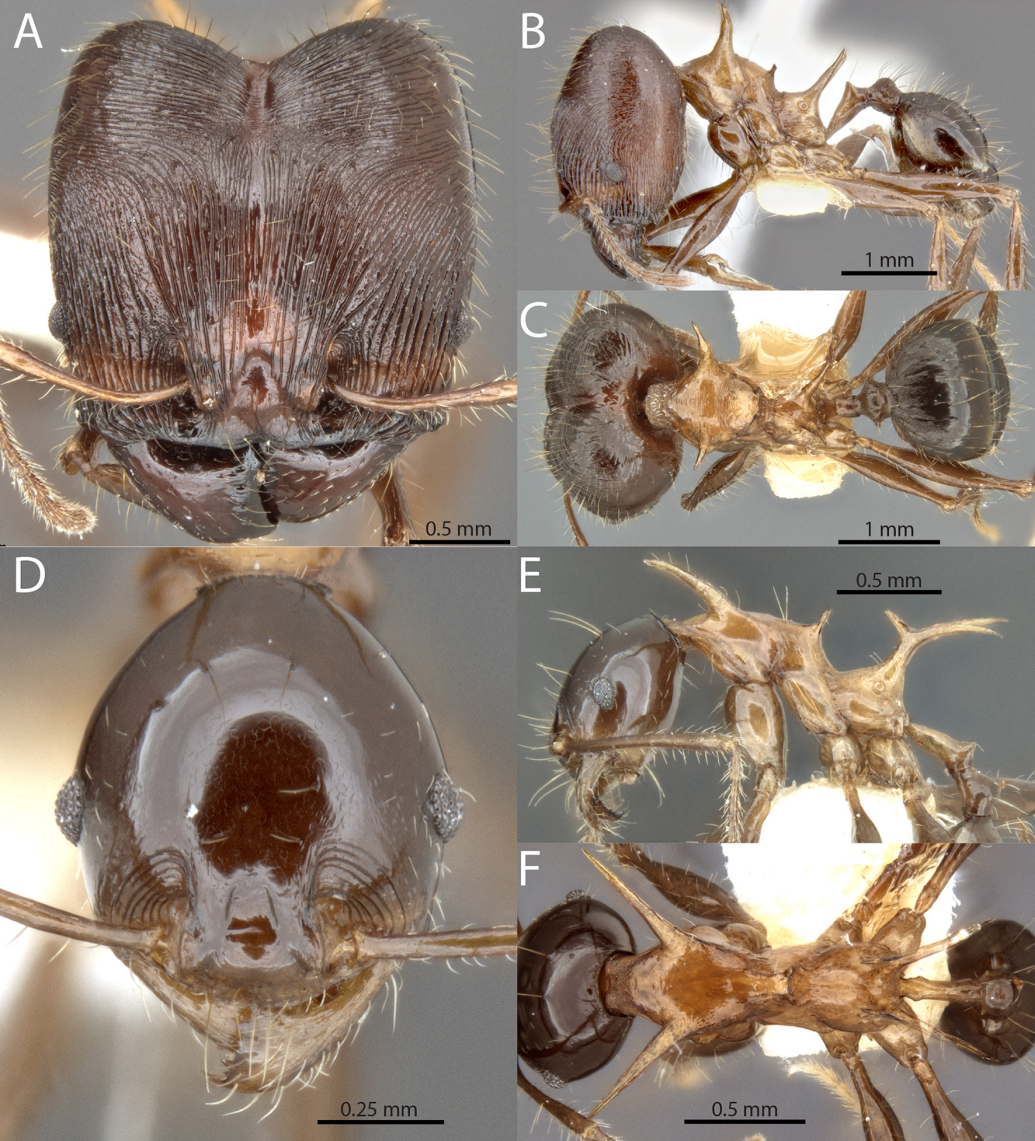
*Pheidole barumtaun* Donisthorpe. Major worker, CASENT0741213: (A) full-face view (B) lateral view (C) dorsal view. Minor worker, LACMENT316356: (D) full-face view (E) profile view (F) dorsal view. From Antweb.org, photographs by April Nobile.

***Pheidole barumtaun*** *Donisthorpe*. (Fig 11)

*Pheidole* (*Pheidolacanthinus*) *barumtaun* Donisthorpe, 1938: 141, figs 1, 2 (s.w.m.). New Guinea [Papua, Jayapura], Cyclops Mountains, Mount Lina, nest in rotten wood, 1070–1370 m, 1936–03 (L.E. Cheesman) [BMNH, 3 syntype majors and 2 syntype minors examined] [28].

= *Pheidole* (*Pheidolacanthinus*) sp. 8 [29]

Major worker. HW 2.10–2.25, HL 2.10–2.22, SL 1.10–1.19, FL 1.62–1.78, EL 0.19–0.24, PSL1 0.61–0.74, PSL3 0.66–0.74, PeL 0.59–0.74, PeW 0.17–0.18, PpW 0.52–0.67, CI 99–105, SI 52–55 (n = 5). Uniformly reddish brown or tricolored with head and gaster reddish brown, mesosoma shining yellowish brown. Head square; posterior margin describing a broad and shallow ‘V’. Head strongly convex in lateral view without any impression of the vertex. Posterolateral lobes separated by a deep median impression giving them a strongly convex appearance. Antennal scrobe absent. Antennal scapes long with erect hairs; strongly curved basally to conform to convexity of head. Frontal carina indistinct; blending with parallel carinulae an undistinguished by strength or length. Anterior medial clypeal margin weakly emarginated. Mandible bidentate apically; striations limited to posterolateral portion. Hypostomal bridge with broad, distinct median tooth; submedian teeth of subequal length to median tooth. Clypeus with strong median carina. All dorsal surfaces of head covered by very fine, parallel, non-intersecting carinulae. Spaces between carinulae weakly foveolate. Carinulae arcuate from anterior clypeal border to frons, converging towards median impression of posterolateral lobes. Carinulae of posterolateral lobes distinctly transverse. Corners of posterolateral lobes shining, but covered with very fine carinulae or striae. Mesosoma armed with long strongly produced pronotal spines, mesonotal spines and propodeal spines. Pronotal spines of subequal length as propodeal spines; directed anterolaterally and becoming downcurved apically. Mesonotal spines approximately eye-length, strongly upturned. Propodeal spines relatively straight, directed posterolaterally. Mesosoma mostly smooth and shining with some transverse striae on dorsum. Petiole relatively long, approximately equal in length to propodeal spine. Petiolar node cuneiform with weakly emarginate vertex. Postpetiole approximately equal in height to petiole; in dorsal view strongly transverse with small lateral projections and weakly sculptured. Basal third of first gastral tergite shagreened.

Minor worker. HW 0.69–0.80, HL 0.80–0.90, SL 1.02–1.18, FL 1.22–1.38, EL 0.15–0.16, ML 1.13–1.28, PSL1 0.53–0.66, PSL2 0.44–0.57, PSL3 0.47–0.62, PeL 0.35–0.45, PeW 0.07–0.13, PpW 0.18–0.23, CI 84–90, SI 146–165 (n = 8). Strongly shining, uniformly reddish brown or tricolored with head and gaster reddish brown, mesosoma shining yellowish brown. Head elongate ovoid, strongly tapering behind the eyes to a narrow posterior margin. Nuchal carinae thin but distinct and forming collar around posterodorsal head margin. Antennal scapes with erect hairs, distinctly surpassing posterior head margin. Head completely smooth except for a few arcuate carinulae between the eyes and mandible. Mesosoma with extremely long spines on the pronotum and propodeum, and shorter spines on the mesonotum. Pronotal spines approximately same length as tibiae; directed anterolaterally and becoming downcurved apically. Mesonotal spines approximately eye-length; spinose to weakly lamellate and flattened; directed posterolaterally and straight. Propodeal spines fused basally into thick upright trunk before diverging; strongly bifurcate, directed posterolaterally and becoming downcurved apically. Petiole very elongate. Petiolar node conical.

The major workers of *Pheidole barumtaun* are separated from those of *P*. *drogon* by the fine carinulae that cover the posterolateral lobes. They are separated from *P*. *viserion* by the less sculptured gastral tergite, less striate mandibles and more arcuate carinulae of the frons. Both major and minor workers of *P*. *barumtaun* are separated from *P*. *viserion* by head and gaster color, which are dark brown in the former and clear yellow in the latter. The minor workers are separated from those of *P*. *drogon* by the narrower head (CI 84–90 *vs*. 89–91), longer legs (FL 1.22–1.38 mm *vs*. 1.07–1.16 mm) and relatively longer antennal scapes (SI 146–165 *vs*. 135–141).

There are, however, some distinct differences between the type series (Cyclops Mts.) and the material collected by Snelling from the Wapoga River Area. The type series workers, both majors and minors, are distinctly tricolored with dark brown heads and gasters that contrast with a yellowish mesosoma. The Wapoga workers are uniformly dark reddish brown. The type series majors also have downcurved propodeal spines, *versus* upturned in the Wapago material. However, there are not clear morphometric differences between these populations, and until further evidence is gathered we consider the variation to be infraspecific.

*Pheidole barumtaun* was named by Donisthorpe after Barumtaun camp where Cheesman made her collection. Among the examined material, workers were collected from the following microhabitats of primary montane forest: under loose bark of log, in and under living bark of recently felled tree, crown of *Pandanus*, decayed stalk in *Pandanus*, in leaf litter. Several of these microhabitats suggest the species might nest and forage in vegetation.

*Material examined*. INDONESIA, Irian Jaya [Papua, Waropen Regency], PT Freeport Concession, -3.14° 136.57°: Wapoga camp, 1998-04-19, 1067m (R.R. Snelling, RRS1998-122); Wapoga camp, 1998-04-21, 1067m (R.R. Snelling, RRS1998-152); Wapoga camp, 1998-04-22, 1067m (R.R. Snelling, RRS1998-160); Wapoga camp, 1998-04-22, 1097m (R.R. Snelling, RRS1998-169); Wapoga camp, 1998-04-24, 1128m (R.R. Snelling, RRS1998-182); Siewa camp, 1998-04-24, 1067m (R.R. Snelling, RRS1998-161); Siewa camp, 1998-04-24, 1128m (R.R. Snelling, RRS1998-184).

**Fig 12:**
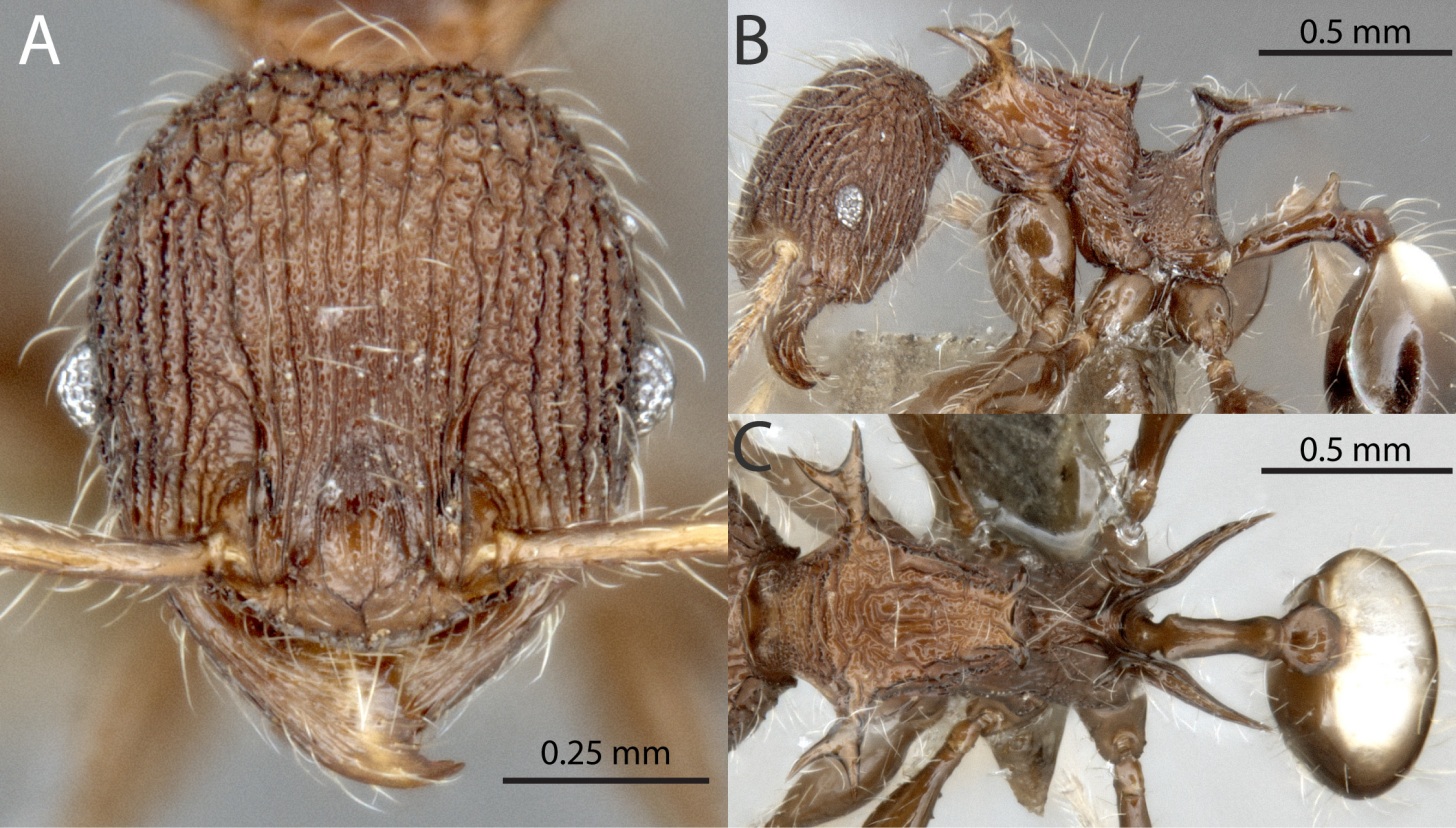
*Pheidole cervicornis* Emery. Minor worker, CASENT0716375: (A) full-face view (B) profile view (C) dorsal view. From Antweb.org, photographs by Masako Ogasawara.

***Pheidole cervicornis*** *Emery*. (Fig 12)

*Pheidole cervicornis* Emery, 1900: 322, pl. 8, fig 25 (w.) New Guinea, Lemien [= Aitape, Papua New Guinea] (L. Biró) [HNHM, 1 syntype worker examined] [30]. Combination in *P*. (*Pheidolacanthinus*): Emery, 1921: 82; Donisthorpe, 1947: 174 [31].

= *Pheidole* (*Pheidolacanthinus*) sp. 5 [29]

= *Pheidole* sp. 1 (det. R.R. Snelling, 2004, New Guinea)

= *Pheidole* sp. 1 (det. R.R. Snelling, 2007, New Guinea)

Major worker. Unknown

Minor worker. HW 0.58–0.64, HL 0.58–0.67, SL 0.61–0.67, FL 0.67–0.78, EL 0.10–0.11, ML 0.76–0.87, PSL2 0.29–0.37, PSL3 0.38–0.47, PeL 0.11–0.35, PeW 0.07–0.09, PpW 0.14–0.17, CI 94–101, SI 101–112. Most dark reddish brown with basal third of first gastral tergite a strongly contrasting white; at least one population from eastern New Guinea a uniform light reddish brown to dark reddish brown and lacking contrasting white portion of gaster. Head ovoid, approximately as wide as long. Posterior head margin relatively broad and flat to weakly concave. Nuchal carinae not visible in full face view. Antennal scapes with erect hairs, distinctly surpassing posterior head margin. Frontal carinae distinct and extending past eye level. Mesosoma with extremely long spines on the pronotum and propodeum, and shorter spines on the mesonotum. Pronotal armament begins as thick basal trunk that diverges into two long anterolaterally pointed prongs. Mesonotal spines approximately eye-length; directed posterolaterally and upturned. Propodeal spines fused basally into thick upright trunk before diverging; strongly bifurcate. Dorsal surface of propodeal spines modified into elongate, sharply margined concavities. Petiole very elongate. Petiolar node acuminate. Dorsal head surface opaque and strongly sculptured. Crenulated longitudinal rugulae become reticulated towards posterior head margin; interspaces strongly punctured. Mandibles weakly striate. Clypeus sculptured. Promesonotal dorsum with crenulated rugae traversing anterior portion and thick longitudinal rugae covering remaining portion. Waist and gaster entirely smooth and shining.

Relatively short-limbed species.

*Pheidole cervicornis*, known only from the minor worker subcaste, is the most distinct member of its eponymous group. The minor workers are the only members of the group which have bifurcated pronotal spines. They are also immediately recognizable by the thickly crenulated rugulae on the head and mesosoma. Additionally, the minor worker has a more circular head with a broad, weakly emarginated posterior margin (*vs*. elongate head with a narrow and flat posterior margin), and all the limbs are relatively shorter. There is some variation in color between specimens collected from western New Guinea (darker and have a contrasting white spot on their gaster), and eastern New Guinea (lighter and lacking white spot). We consider this infraspecific variation until additional collections prove otherwise. Aside from one nest collected from beneath a stone by Wilson (20), *Pheidole cervicornis* has only been recorded from stray foragers collected from the ground, in leaf litter, on logs, and in logs. It is the most widely collected species of the group, occurring along the northern coast of New Guinea, and also occupies the lowest elevation range (30–800 m). The species also appears to be more tolerant of disturbance than its close relatives, and has been collected in secondary and disturbed forest habitats.

*Material examined*. INDONESIA, Papua: PT Freeport Concession, Siewa Camp, -3.04° 136.38°, 61m, 1998-04-10 (R.R. Snelling, RRS98-63); PT Freeport Concession, Siewa Camp, -3.04° 136.38°, 61m, 1998-04-15 (R.R. Snelling); 8.1 km ENE Gesa, Gesa river, Mambermo Basin, -2.08° 137.46°, 40m, 2007-05-01 (R.R. Snelling, RRS07-019, RRS07-025, RRS07-026, RRS07-027). PAPUA NEW GUINEA. East Sepik, 3km S. Wewak, -3.61667° 143.61667°, 400m, 1989-02-15 (P.S. Ward, PSW10203-4); Madang, 5km SW Mt. Uluman, Karkar Island, -4.6833° 145.9500°, 800m, 1989-02-05 (P.S. Ward, PSW10142-28); Madang, Ohu village, -5.23333° 145.68333°, 200m, 1999-03-04 (E.M. Sarnat, EMS934); Madang, Ohu village, -5.26° 145.68°, 150m, 2002-01-03 (M. Janda); Madang, Baitabag, -5.143° 145.772°, 30m, 2009-11-14 (M. Janda *et al*.); Madang, Baitabag, -5.1333° 145.7833°, 50m, 2010-11-03 (M. Janda).

**Fig 13:**
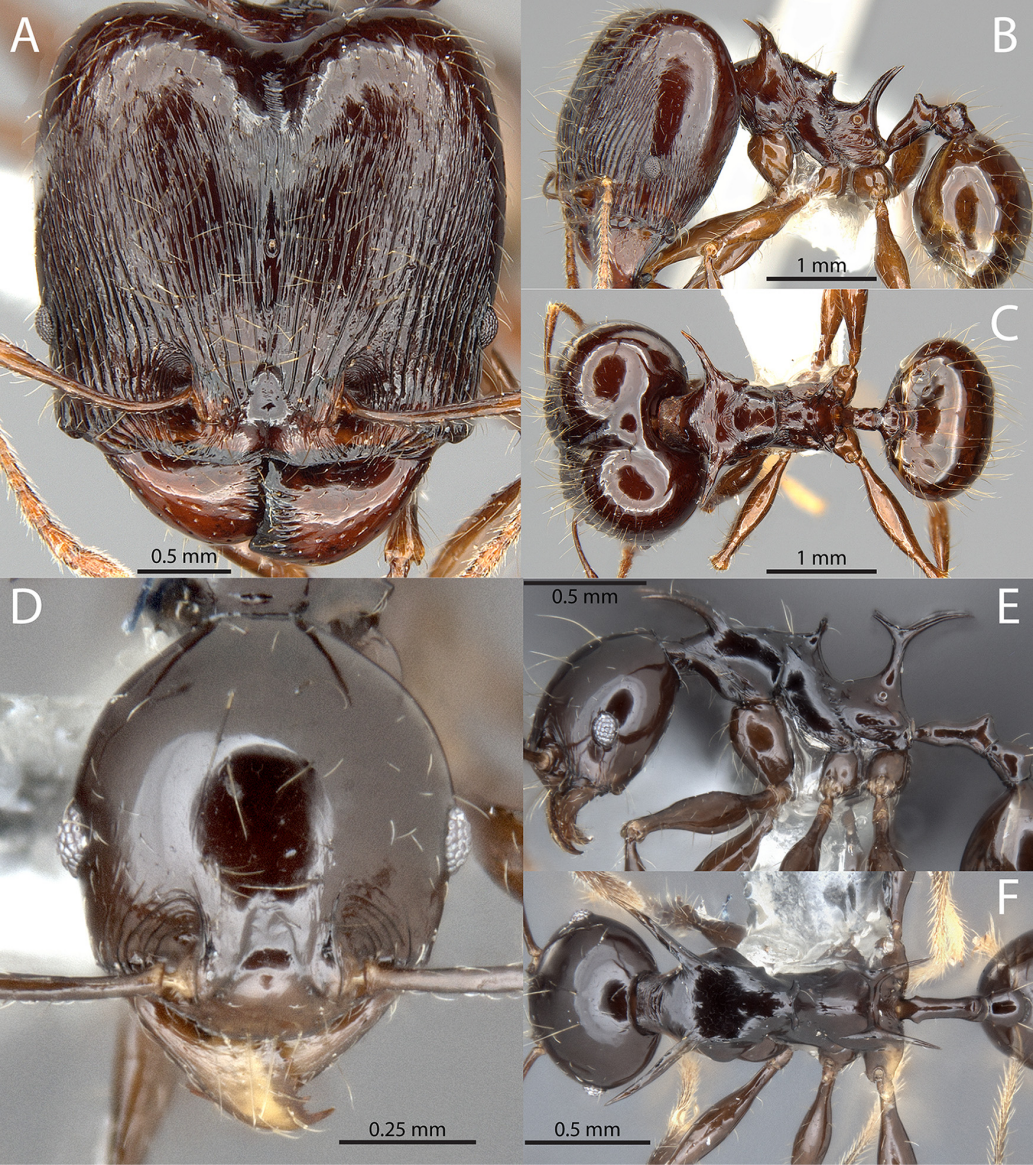
*Pheidole drogon* sp. nov. Major worker, holotype, CASENT0716380: (A) full-face view (B) lateral view (C) dorsal view. Minor worker, paratype, CASENT0753009: (D) full-face view (E) profile view (F) dorsal view. Photographs by Masako Ogasawara.

***Pheidole drogon*** *Sarnat*, *Fischer & Economo* ***sp*. *nov*.** [urn:lsid:zoobank.org:act:4430D4A8-263C-412A-BAAC-2D989422F363] (Fig 13)

Holotype. Papua New Guinea, Morobe, 11km E Baiyer River Sanctuary, 5.5000° S, 144.2667° E, 1900 m, 24.vi.1980, P. S. Ward, PSW4575-7, montane rainforest, at tuna bait, (major worker, dry pinned, USNM, specimen code CASENT0716380). Paratypes (same data as holotype): 1 minor worker (CASENT0753009, USNM); 2 minor workers, 1 major worker (CASENT0716381, USNM).

= *Pheidole* sp. EMSM118

= *Pheidole* sp. EMSM119

Major worker. HW 2.16–2.19, HL 2.08–2.14, SL 1.07–1.08, FL 1.61–1.66, EL 0.18–0.19, ML 1.68, PSL1 0.63–0.70, PSL2 0.61, PeL 0.61–0.64, PeW 0.16–0.17, PpW 0.56–0.58, CI 103–104, SI 49 (n = 2). Color uniformly reddish brown, coxae and legs a weakly contrasting yellowish brown. Head square; posterior margin describing a broad and shallow ‘V’. Head moderately convex in lateral view without any impression of the vertex. Posterolateral lobes separated by a moderate median impression. Antennal scrobe absent. Antennal scapes long with erect hairs; moderately curved basally to conform to convexity of head. Frontal carina indistinct; blending with parallel carinulae an undistinguished by strength or length. Anterior medial clypeal margin weakly emarginated. Mandible bidentate apically; striations restricted to basal portion. Hypostomal bridge with broad, distinct median tooth; submedian teeth of subequal length to median tooth. Clypeus mostly smooth and shining, median carina weak to absent. All surfaces of head covered by fine, parallel, non-intersecting carinulae. Carinulae irregularly longitudinal from anterior clypeal border to frons, diverging weakly towards posterolateral corners and terminating before reaching posterior margin. Posterolateral lobes with smooth and shining corners; lateral and ventral portion of lobes smooth and shining. Mesosoma armed with long strongly produced pronotal spines and propodeal spines; mesonotum armed with broad and flattened lamellate lobes. Pronotal spines of subequal length as propodeal spines; directed anterolaterally and becoming downcurved apically. Propodeal spines relatively straight, directed posterolaterally, becoming weakly downcurved apically. Pronotal dorsum smooth to weakly transversely striate; mesosoma otherwise mostly smooth and shining. Petiole relatively long, approximately equal in length to propodeal spine. Petiolar node cuneiform with weakly emarginate vertex. Postpetiole approximately equal in height to petiole; in dorsal view mostly smooth and shining with several transverse striations on posterior portion and moderate lateral projections. Entire gaster smooth and shining.

Minor worker. HW 0.67–0.73, HL 0.75–0.81, SL 0.94–1.00, FL 1.07–1.16, EL 0.12–0.14, ML 1.06–1.15, PSL1 0.45–0.52, PSL2 0.43–0.49, PSL3 0.36–0.42, PeL 0.35–0.39, PeW 0.07–0.08, PpW 0.15–0.17, CI 89–91, SI 135–141 (n = 8). Strongly shining reddish brown, coxae and legs a weakly contrasting yellowish brown. Head elongate ovoid, tapering weakly behind the eyes to a narrow posterior margin. Nuchal carinae thin but distinct and forming collar around posterodorsal head margin. Antennal scapes with erect hairs, distinctly surpassing posterior head margin. Head completely smooth except for a few arcuate carinulae between the eyes and mandible. Mesosoma with extremely long spines on the pronotum and propodeum, and shorter spines on the mesonotum. Pronotal spines emarginate; approximately same length as tibiae; directed anterolaterally and becoming downcurved apically. Mesonotal spines approximately eye-length; directed posterolaterally and straight. Propodeal spines fused basally into thick upright trunk before diverging; strongly bifurcate, directed posterolaterally and becoming downcurved apically. Petiole very elongate. Petiolar node conical.

The major workers of *Pheidole drogon* are distinguished from those of all other *cervicornis*-group species by the posterolateral lobe and gastral tergite, both of which are glossy and free of sculpture or shagreening. The minor workers are difficult to distinguish from those of *P*. *barumtaun*, and readers are referred to the discussion of that species for differentiating characteristics. The species is only known from the type locality where it was collected in montane rainforest on low vegetation and also recruiting to a tuna bait.

Etymology: The species name refers to Drogon, the black-colored dragon of Daenerys Targaryen, a fictional character from the George R. R. Martin’s novel A Song of Ice and Fire. The name is a noun in apposition and thus invariable.

*Material examined*. PAPUA NEW GUINEA, Morobe, 11km E Baiyer River Sanctuary, -5.5000° 144.2667°, 1900 m: 1980-06-23 (P.S. Ward, PSW4528); 1980-06-24 (P.S. Ward, PSW4553-3).

**Fig 14:**
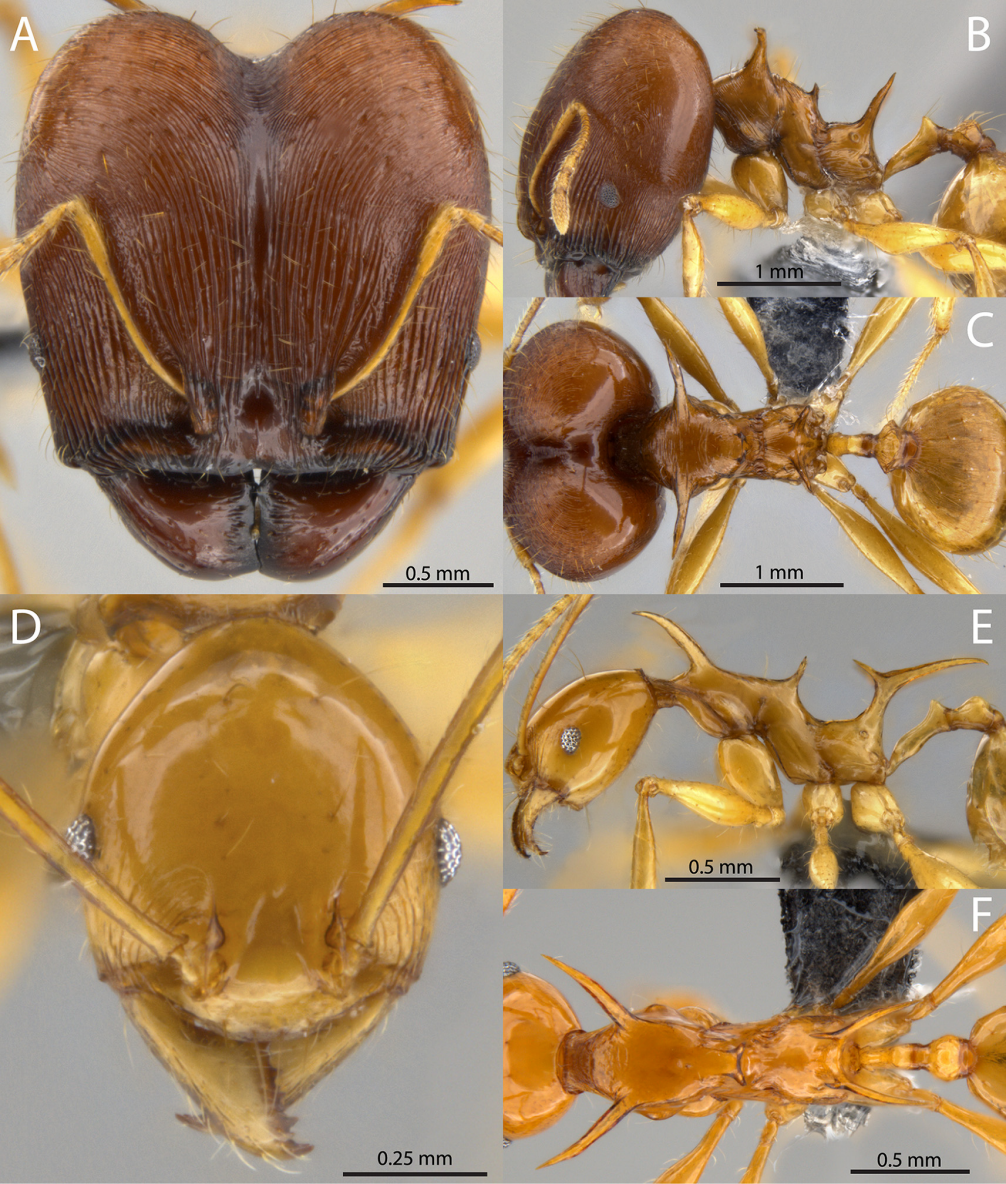
*Pheidole viserion* sp. nov. Major worker, holotype, CASENT0219462: (A) full-face view (B) lateral view (C) dorsal view. Minor worker, paratype, CASENT0282331: (D) full-face view (E) profile view (F) dorsal view. Photographs by Masako Ogasawara.

***Pheidole viserion*** *Sarnat*, *Fischer & Economo* ***sp*. *nov*.** [urn:lsid:zoobank.org:act:80B52A26-7470-4D15-9A3C-BCCE923FED65] (Fig 14)

Holotype. Papua New Guinea, Southern Highlands, Kutubu area, Mubi River, Kantobo Village, 6.726° S, 143.567° E, 451 m, 9.ii.2008, M. Janda & S. Ibalim, MJ13993, limestone hill (major worker, dry pinned, USNM, specimen code CASENT0219462). Paratypes (same data as holotype): 3 minor workers, USNM (CASENT0282331, CASENT0199517, CASENT0282332).

= *Pheidole* sp. EPEM109 (nr. *barumtaun*) [1]

= *Pheidole* sp. EPEM109 (nr. *barumtaun*) [8]

= *Pheidole* sp. PG07 [7]

Major worker. HW 2.09–2.13, HL 2.06–2.11, SL 1.05–1.08, FL 1.60–1.68, EL 0.18–0.20, ML 1.50–1.67, PSL1 0.57–0.68, PSL3 0.70–0.74, PeL 0.59–0.62, PeW 0.14, PpW 0.43–0.46, CI 99–101, SI 50–52 (n = 3). Color uniformly shining yellow, major sometimes with darker yellowish brown head. Head square; posterior margin describing a broad and shallow ‘V’. Head strongly convex in lateral view without any impression of the vertex. Posterolateral lobes separated by a deep median impression giving them a strongly convex appearance. Antennal scrobe absent. Antennal scapes long with erect hairs; strongly curved basally to conform to convexity of head. Frontal carina indistinct; blending with parallel carinulae an undistinguished by strength or length. Anterior medial clypeal margin weakly emarginated. Mandible bidentate apically; entirely striate. Hypostomal bridge with broad, distinct median tooth; submedian teeth of subequal length to median tooth. Clypeus with strong median carina. All surfaces of head covered by fine, parallel, non-intersecting carinulae. Carinulae longitudinal from anterior clypeal border to frons, then diverging laterally and becoming finer. Carinulae of posterolateral lobes arcuate to distinctly transverse. Mesosoma armed with long strongly produced pronotal spines, mesonotal spines and propodeal spines. Pronotal spines of subequal length as propodeal spines; directed anterolaterally and becoming downcurved apically. Mesonotal spines approximately eye-length, strongly upturned. Propodeal spines relatively straight, directed posterolaterally, becoming weakly downcurved apically. Pronotal dorsum transversely striate; mesosoma otherwise mostly smooth and shining. Petiole relatively long, approximately equal in length to propodeal spine. Petiolar node cuneiform with weakly emarginate vertex. Postpetiole approximately equal in height to petiole; in dorsal view strongly transverse with small lateral projections and weakly sculptured. Entire first gastral tergite longitudinally striate.

Minor worker. HW 0.62–0.68, HL 0.75–0.83, SL 1.04–1.12, FL 1.23–1.44, EL 0.12–0.15, ML 1.09–1.24, PSL1 0.53–0.63, PSL2 0.50–0.60, PSL3 0.45–0.60, PeL 0.38–0.44, PeW 0.07–0.10, PpW 0.16–0.19, CI 81–87, SI 162–169 (n = 10). Strongly shining yellow. Head elongate ovoid, tapering behind the eyes to a narrow posterior margin. Nuchal carinae thin but distinct and forming collar around posterodorsal head margin. Antennal scapes with erect hairs, distinctly surpassing posterior head margin. Head completely smooth except for a few arcuate carinulae between the eyes and mandible. Mesosoma with extremely long spines on the pronotum and propodeum, and shorter spines on the mesonotum. Pronotal spines approximately same length as tibiae; directed anterolaterally and becoming downcurved apically. Mesonotal spines approximately eye-length; directed posterolaterally and straight. Propodeal spines fused basally into thick upright trunk before diverging; strongly bifurcate, directed posterolaterally and becoming downcurved apically. Petiole very elongate. Petiolar node conical.

*Pheidole viserion* is the only member of the *cervicornis* group that is uniformly yellow in color. The species is most similar to *P*. *barumtaun*, but the majors of *P*. *viserion* can be distinguished by the more striate mandibles and first gastral tergite, and cephalic sculpture. The minor workers are quite similar to those of *P*. *barumtaun*, and are best differentiated by the uniform yellow color.

*Pheidole viserion* has been collected from three sites in Papua New Guinea from montane primary forests and from a lowland habitat transitioning between primary and secondary forest. In addition to being found in the leaf litter, it was also found foraging in a hollow trunk above the ground.

Etymology: The species name refers to Viserion, the cream and gold colored dragon of Daenerys Targaryen, a fictional character from the George R. R. Martin’s novel A Song of Ice and Fire. The name is a noun in apposition and thus invariable.

*Material examined*. PAPUA NEW GUINEA. Chimbu, 11 km E Haia airstrip, -6.71667° 145.10000°, 900 m, 1999-02-10 (E.M. Sarnat, EMS687); East Highlands, Simbu, -6.72° 145.09°, 1075 m, 2001 (K. Sagata, EX1P23N28); Morobe, Finisterre Mts., Yawan, Yawan-Ubi-camp, -6.16271° 146.839°, 1600 m, 2010-10-29 (M. Janda MJ13587).

## Supporting Information

S1 Fig *Pheidole barumtaun* Donisthorpe volumetric surface model (major worker, CASENT0709598). If viewed with Adobe Acrobat Reader (version 8 or higher), the interactive 3D-mode can be activated after trusting the document by clicking on the image, allowing the user to rotate, move and magnify the model.
(PDF)

S2 Fig *Pheidole barumtaun* Donisthorpe volumetric surface model (minor worker, CASENT0741213). If viewed with Adobe Acrobat Reader (version 8 or higher), the interactive 3D-mode can be activated after trusting the document by clicking on the image, allowing the user to rotate, move and magnify the model.
(PDF)

S3 Fig *Pheidole cervicornis* Emery volumetric surface model (minor worker, CASENT0282330). If viewed with Adobe Acrobat Reader (version 8 or higher), the interactive 3D-mode can be activated after trusting the document by clicking on the image, allowing the user to rotate, move and magnify the model.
(PDF)

S4 Fig *Pheidole drogon* sp. nov. volumetric surface model (major worker, holotype, CASENT0716380). If viewed with Adobe Acrobat Reader (version 8 or higher), the interactive 3D-mode can be activated after trusting the document by clicking on the image, allowing the user to rotate, move and magnify the model.
(PDF)

S5 Fig *Pheidole drogon* sp. nov. volumetric surface model (minor worker, paratype, CASENT0753009). If viewed with Adobe Acrobat Reader (version 8 or higher), the interactive 3D-mode can be activated after trusting the document by clicking on the image, allowing the user to rotate, move and magnify the model.
(PDF)

S6 Fig *Pheidole viserion* sp. nov. volumetric surface model (major worker, holotype, CASENT0219462). If viewed with Adobe Acrobat Reader (version 8 or higher), the interactive 3D-mode can be activated after trusting the document by clicking on the image, allowing the user to rotate, move and magnify the model.
(PDF)

S7 Fig *Pheidole viserion* sp. nov. volumetric surface model (minor worker, paratype, CASENT0282331). If viewed with Adobe Acrobat Reader (version 8 or higher), the interactive 3D-mode can be activated after trusting the document by clicking on the image, allowing the user to rotate, move and magnify the model.
(PDF)

S1 Vid *Pheidole barumtaun* Donisthorpe volumetric surface rendering rotational video (major worker, CASENT0709598). (MPG)

S2 Vid *Pheidole barumtaun* Donisthorpe volumetric surface rendering rotational video (minor worker, CASENT0741213). (MPG)

S3 Vid *Pheidole cervicornis* Emery volumetric surface rendering rotational video (major worker, CASENT0282330). (MPG)

S4 Vid *Pheidole drogon* sp. nov. volumetric surface rendering rotational video (major worker, holotype, CASENT0716380). (MPG)

S5 Vid *Pheidole drogon* sp. nov. volumetric surface rendering rotational video (minor worker, paratype, CASENT0753009). (MPG)

S6 Vid *Pheidole viserion* sp. nov. volumetric surface rendering rotational video (major worker, holotype, CASENT0219462). (MPG)

S7 Vid *Pheidole viserion* sp. nov. volumetric surface rendering rotational video (minor worker, paratype, CASENT0282331). (MPG)

S8 Vid False-color volume rendering video of virtually dissected mesosoma (*Pheidole viserion*, major worker, holotype, CASENT0219462). Muscle fibers are colored in red.
(MPG)

S9 Vid Orthoslice virtual dissection of mesosoma (*Pheidole barumtaun*, major worker, CASENT0741213). Muscle fibers are colored in red.
(MP4)
