# Supplementary figures and images for: Inordinate Spinescence: Taxonomic Revision and Microtomography of the Pheidole cervicornis Species Group (Hymenoptera, Formicidae)
Source: PLoS One. 2016 Jul 27;11(7):e0156709. doi: 10.1371/journal.pone.0156709 (PMC4963106; doi:10.1371/journal.pone.0156709)

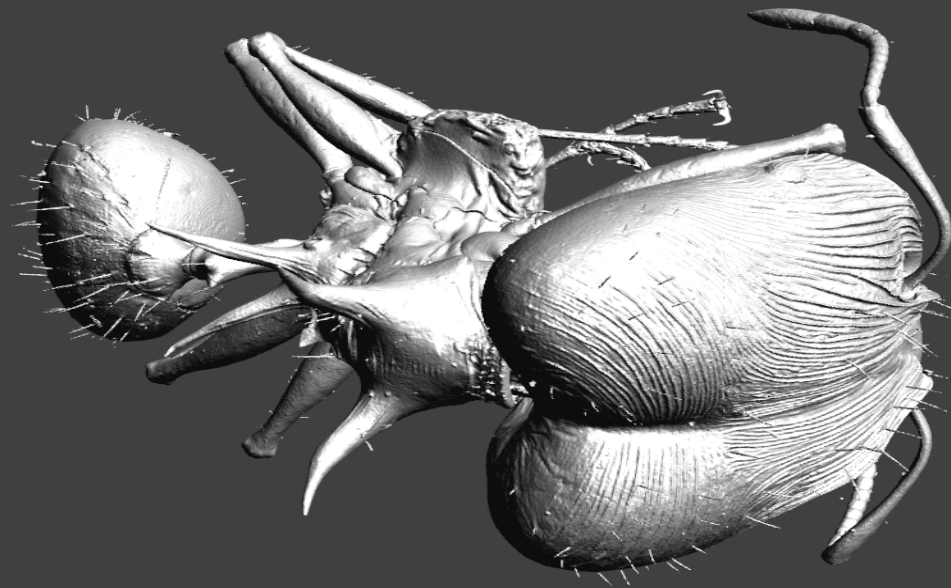

*Pheidole barumtaun*  
major worker CASENT0709598

Supplement: S1 Fig — If viewed with Adobe Acrobat Reader (version 8 or higher), the interactive 3D-mode can be activated after trusting the document by clicking on the image, allowing the user to rotate, move and magnify the model. (PDF) [file pone.0156709.s001.pdf]

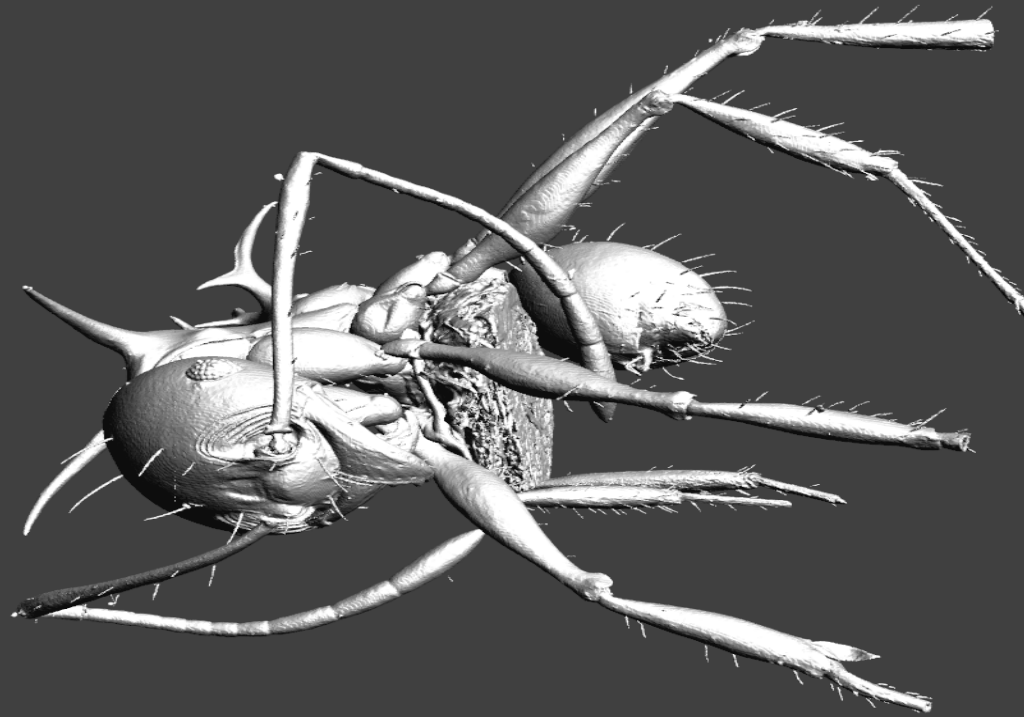

*Pheidole barumtaun*  
minor worker CASENT0741213

Supplement: S2 Fig — If viewed with Adobe Acrobat Reader (version 8 or higher), the interactive 3D-mode can be activated after trusting the document by clicking on the image, allowing the user to rotate, move and magnify the model. (PDF) [file pone.0156709.s002.pdf]

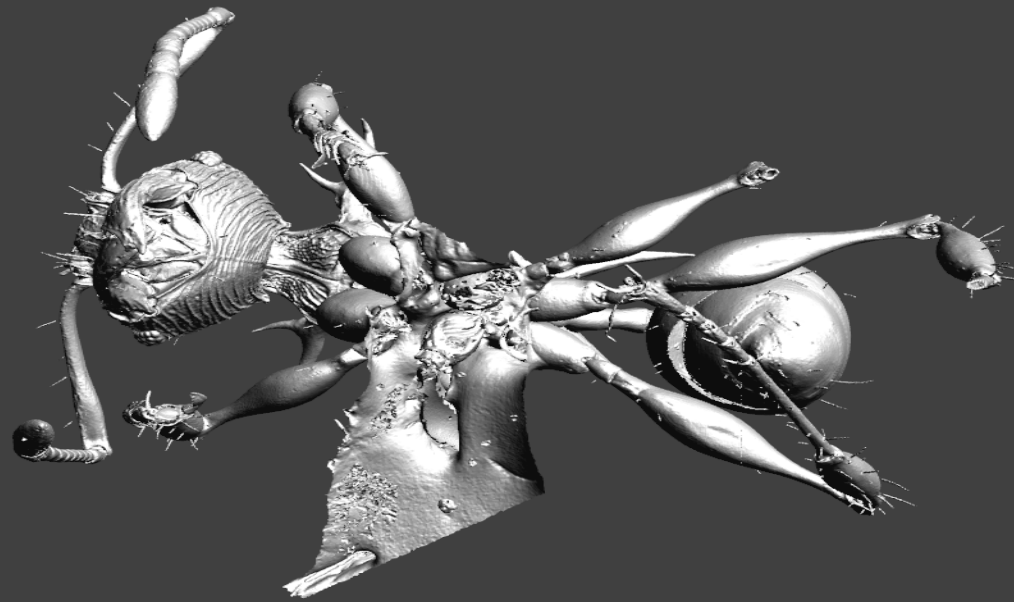

*Pheidole cervicornis*  
minor worker CASENT0282330

Supplement: S3 Fig — If viewed with Adobe Acrobat Reader (version 8 or higher), the interactive 3D-mode can be activated after trusting the document by clicking on the image, allowing the user to rotate, move and magnify the model. (PDF) [file pone.0156709.s003.pdf]

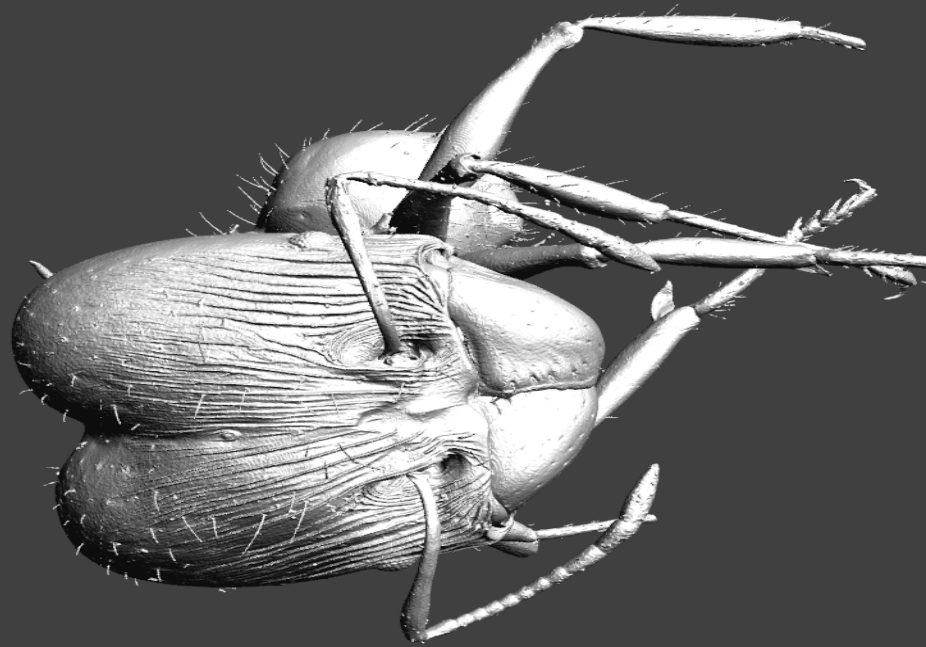

*Pheidole drogon*  
major worker CASENT0716380

Supplement: S4 Fig — If viewed with Adobe Acrobat Reader (version 8 or higher), the interactive 3D-mode can be activated after trusting the document by clicking on the image, allowing the user to rotate, move and magnify the model. (PDF) [file pone.0156709.s004.pdf]

Sarnat EM, Fischer G, Economo EP (2016)  
Inordinate spinescence

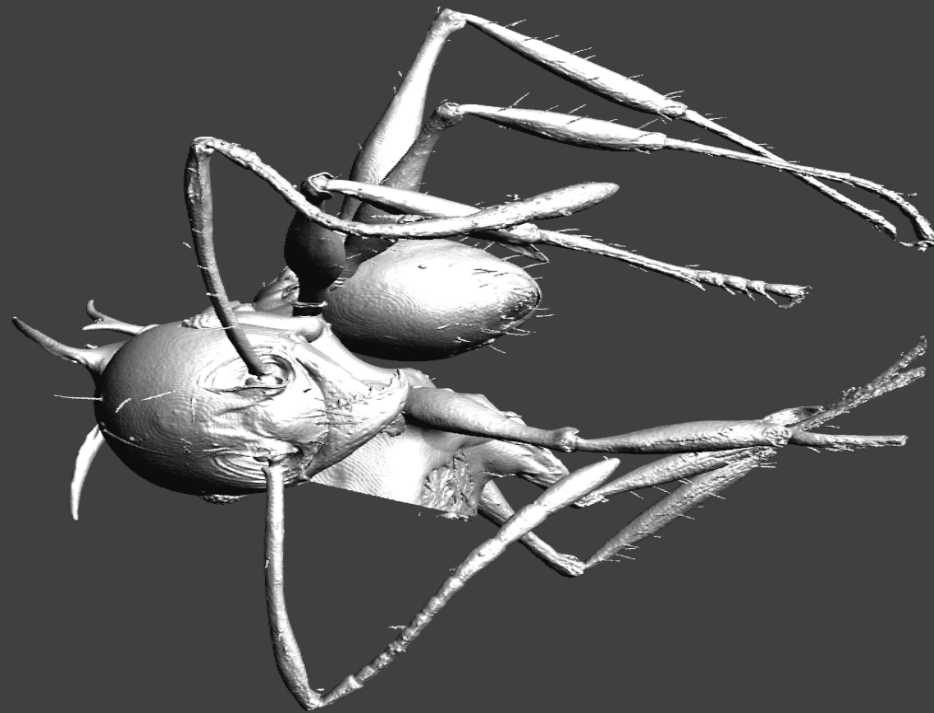

*Pheidole drogon*  
minor worker CASENT0753009

Supplement: S5 Fig — If viewed with Adobe Acrobat Reader (version 8 or higher), the interactive 3D-mode can be activated after trusting the document by clicking on the image, allowing the user to rotate, move and magnify the model. (PDF) [file pone.0156709.s005.pdf]

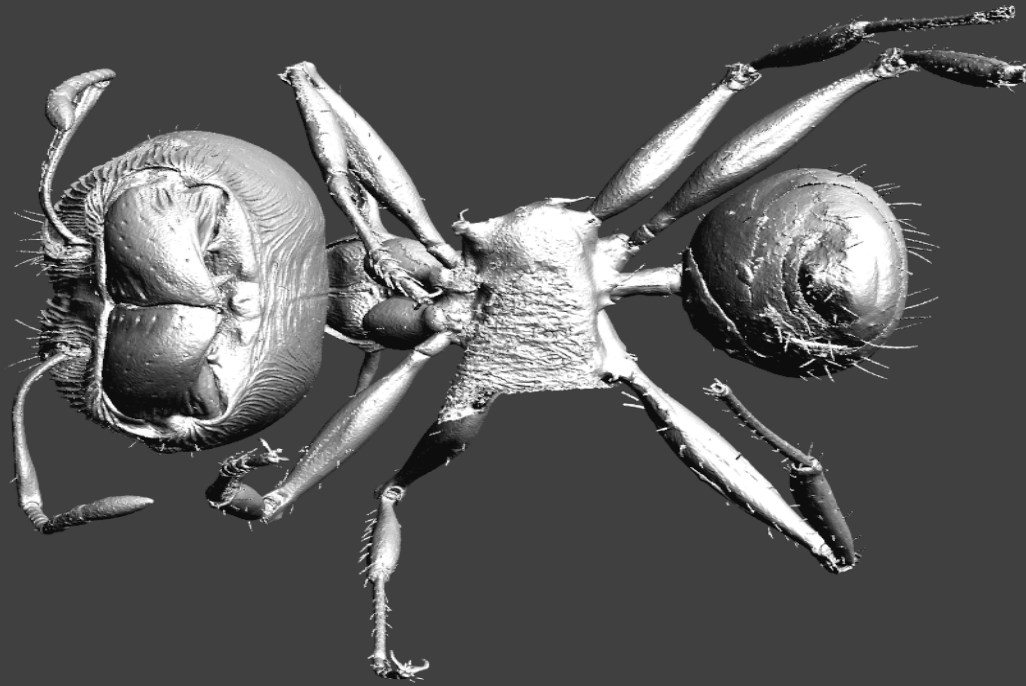

*Pheidole viserion*  
major worker CASENT0219462

Supplement: S6 Fig — If viewed with Adobe Acrobat Reader (version 8 or higher), the interactive 3D-mode can be activated after trusting the document by clicking on the image, allowing the user to rotate, move and magnify the model. (PDF) [file pone.0156709.s006.pdf]

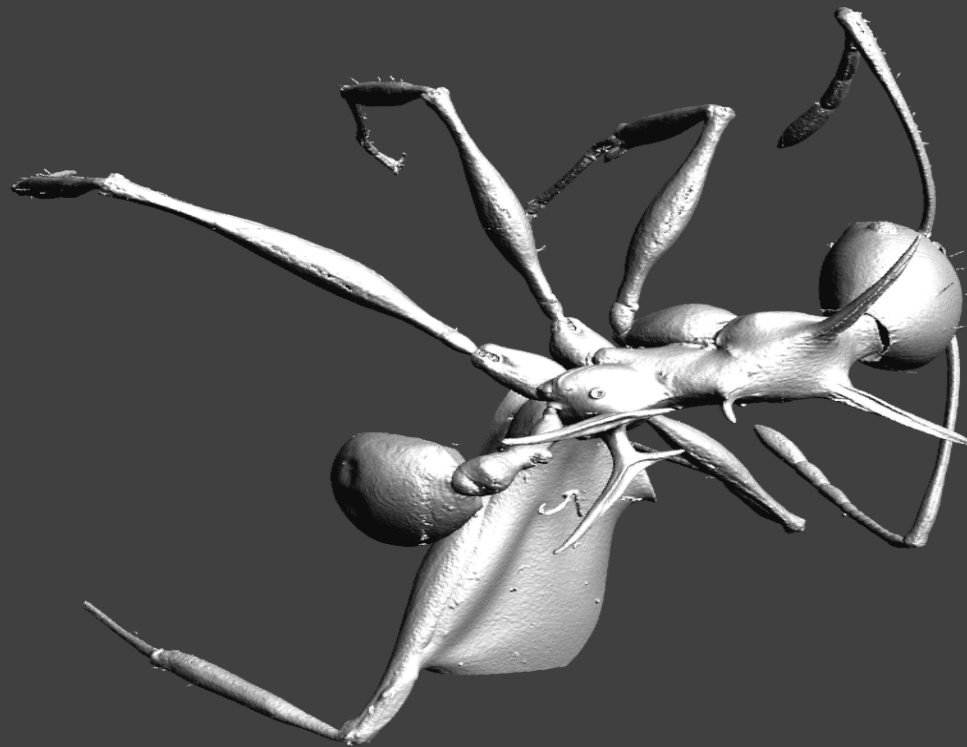

*Pheidole viserion*  
minor worker CASENT0282331

Supplement: S7 Fig — If viewed with Adobe Acrobat Reader (version 8 or higher), the interactive 3D-mode can be activated after trusting the document by clicking on the image, allowing the user to rotate, move and magnify the model. (PDF) [file pone.0156709.s007.pdf]
